# A Simulation-Free Topological Basis for Building Compact Koopman Models of Protein Folding

**DOI:** 10.1021/acs.jcim.6c01905

**Published:** 2026-09-01

**Authors:** Ziad Fakhoury, Gabriele C. Sosso, Scott Habershon

**Affiliations:** Department of Chemistry, 2707 University of Warwick, Coventry CV4 7AL, United Kingdom

## Abstract

Unravelling protein-folding mechanisms and kinetics is a key challenge to biochemical science. The variational approach for Markov processes (VAMP) is a powerful tool to build Markov Models that capture key kinetic and structural information despite the conformational complexity and long time scales associated with protein folding. However, VAMP-based Markov models use data from exhaustive molecular dynamics (MD) simulations to construct an underlying basis set describing the “coarse-grained” kinetics; the same MD data can be used to predict transition probabilities between partitioned configuration space, enabling the extraction of folding time scales and mechanism. Here, we propose an alternative strategy for Markov model construction that does not rely on extensive, computationally demanding MD data for configuration space partitioning. Specifically, we show that graph-driven sampling (GDS) can generate a complete “landscape” of intermediate contact-maps linking unfolded and folded protein conformations; importantly, extensive MD simulations are not required in GDS. When combined with a physically intuitive shortest-contact-hop metric to discriminate different intermediate states, GDS mapping of protein-folding configuration space generates a reliable “structurally aware” partitioning for VAMP model construction. To demonstrate this strategy, we show that a GDS-constructed Markov model variationally improves folding time scale estimates for all-atom models of the WW domain protein—and has the additional advantage of easily resolving kinetic traps in the folding landscape that have proven challenging to confirm otherwise. Together, the combination of GDS and VAMP opens a new route toward rapid characterization of protein-folding intermediates and kinetics traps to help address frontier challenges such as protein misfolding, aggregation, and protein design.

## Graphical abstract

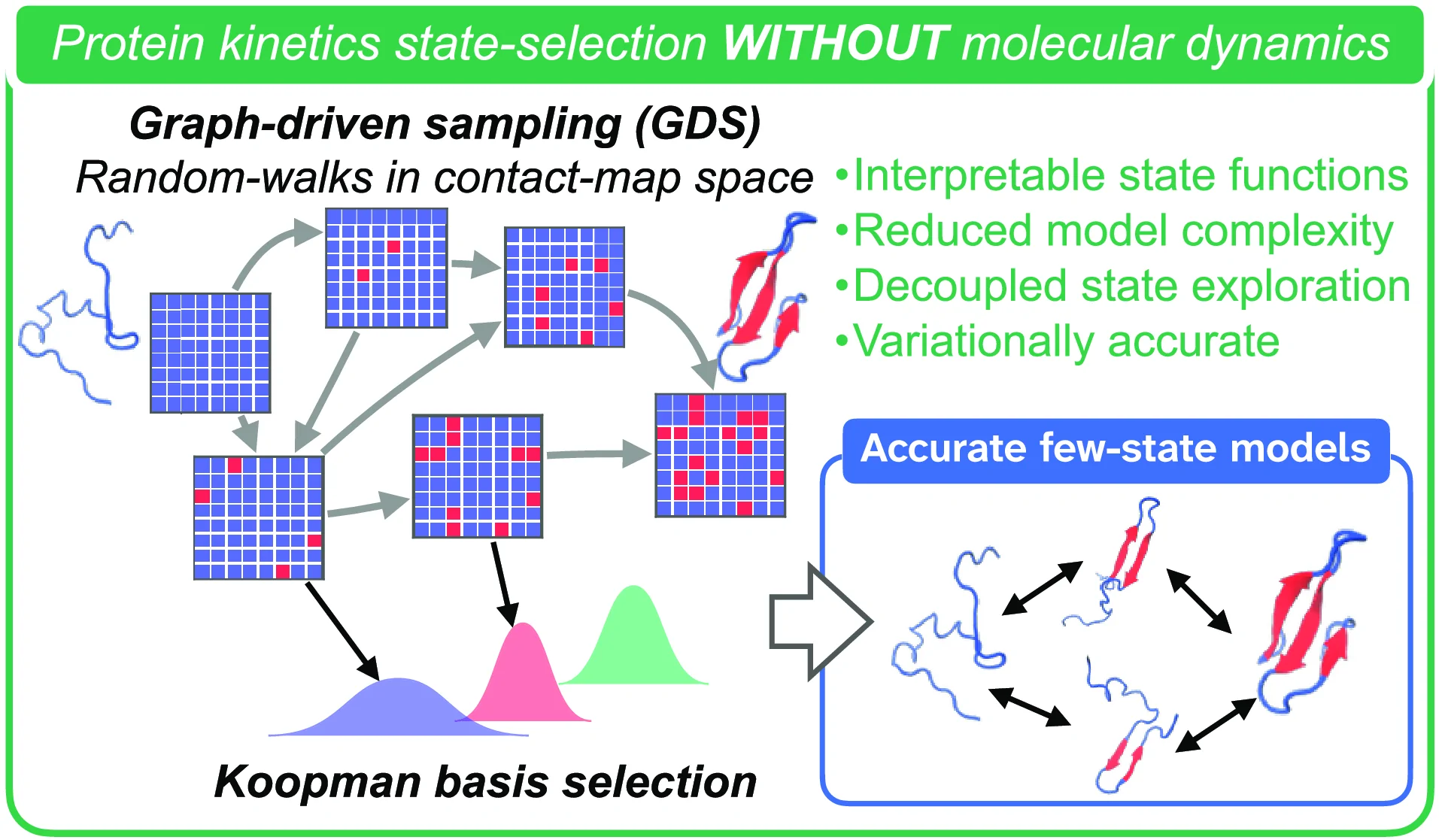

## Introduction

Protein structure prediction has recently been successfully addressed with the development of deep learning techniques using mined protein-sequence alignment data.^1,2^ On the other hand, understanding the dynamic pathways by which proteins obtain their folded native state remains an important open problem that is critical for better understanding of important biophysical phenomena such as ligand-binding interactions, protein–protein interfaces, protein misfolding, and allosteric regulation.^3^ “Foldability” is also an often-neglected feature in contemporary computational protein design workflows—a drawback that could be addressed with better access to computational insights into folding mechanism and kinetics.^4,5^

In general, the time scale separation between fast local fluctuations encoded in atomistic models of proteins and the slow relevant conformational transitions gives rise to a rare event problem that hinders the computational study of protein-folding. Advances in both algorithms and hardware have provided some limited access to extremely long molecular dynamics (MD) trajectories of folding events, with a particularly prominent example being the microsecond-long MD simulations generated for several fast folding proteins using the custom-made ANTON computing architecture.^6^ However, extracting kinetic and mechanistic properties from this collection of data—spanning over 1 ms with MD snapshots taken every 2.5 ps for systems containing up to 561 protein atoms (alongside ≈ 4300 water molecules)—is a daunting task, albeit an important one for the study of protein dynamics.

Markov State Models (MSMs) have emerged as a powerful frameworkk for modeling protein folding dynamics by discretizing conformational space into metastable states, and subsequently estimating the transition probabilities between them.^7–10^ By leveraging existing collections of MD simulations, MSMs are linear models of protein dynamics which enable an efficient and straightforward reconstruction of folding pathways and the computation of long-time scale kinetics. Constructing accurate MSMs requires careful state discretization and sufficient *a priori* MD sampling, making this approach sensitive to a number of user-specified parameters as well as the nature of the available MD data.^11,12^

To address the choice of state discretization, the variational approach to conformational dynamics (VAC) and the more general variational approach for Markov processes (VAMP) were introduced as frameworks to optimize MSMs by maximizing a variational score, offering a principled way to compare different discretizations.^13,14^ Furthermore, these variational strategies allow for the generalization of MSMs to Markov models defined with “soft states”—functions that act as a basis to express conformational probability densities and dynamical models. This paved the way for neural networks—VAMPNets—to replace traditional state selection methods with neural-network-parametrized “soft states” that maximize the variational score, improving state-space resolution and kinetic accuracy.^15^ However, these variational approaches still have a high dependency on MD data generation—a severe computational bottleneck that hinders their broader applicability. MD simulations for fitting both discrete-state MSMs and “soft state” Markov models is an active area of research, with current approaches making use of active, adaptive and enhanced sampling techniques.^16–21^

Recently, we have proposed a simulation strategy to generate protein folding pathways using a contact-map-based representation, termed graph-driven sampling (GDS).^22,23^ Crucially, we are able to generate a folding pathway ensemble without performing extensive MD simulations or using predefined order parameters. The GDS approach directly identifies and characterizes those regions of the protein structural landscape that are explored in the dynamic folding process—information that could provide an alternative route to constructing a suitable state definition for a Markov model.

In this work, we hypothesize that the intermediate states defined by the contact-map–based folding trajectories available from GDS can inform the construction of an ideal basis set for building a Markov model—without resorting to any *a priori* MD simulations. Specifically, each contact map in our GDS trajectories represents a distinct “state” of the protein that reflects significant conformational changes in the protein structure. As the protein folds, the sequential transformation captured in its contact map traces a pathway through a reduced conformational space that is both physically interpretable and computationally tractable. By using this set of states as a basis, a Markov model can be constructed to (i) propagate probability densities defined on contact map states; (ii) identify slow transition modes in the contact map representation, and; (iii) quantify kinetic parameters such as relaxation time scales and metastable state residence times.

The key advantage with this approach is that, unlike the popular VAMPNet^15^ and the earlier MSM approach,^9^ a basis for a Markov Model can be built without any MD data *a priori*. Generating such data usually incurs a considerable cost; in fact, VAMPNets are currently trained on an initial smaller MD data set that are then used to seed further MD simulations in an iterative fashion.^16,18–21^ However, given that our approach identifies a suitable basis without the need of any MD data to begin with, it can enable either strategic use of enhanced MD simulations^24^ or rate estimates from path sampling and optimization techniques.^25,26^ This would represent a paradigm shift in how future Markov models for protein folding are constructed.

The proposed approach therefore bridges the gap between discrete structural protein conformational transitions and continuous kinetic modeling of protein dynamics. The discrete contact-map representation used in GDS circumvents many challenges encountered in direct MD simulations. For example, the demand for long simulation times to sample rare transitions and the difficulty in selecting an appropriate order parameter are circumvented in GDS by providing an alternative “coordinate system” in which the most significant folding events are emphasized. In turn, as shown below, the Markov model built upon this basis offers a systematic and quantitative description of the folding kinetics that is not only predictive but also interpretable in terms of underlying molecular events.

Furthermore, the residue–residue graph representation of a protein conformation, known as a contact map, is thought to encode the structurally relevant information defining the protein’s topology. They have historically been a key representation used in seminal works in protein folding.^27–29^ Therefore, contact maps, as employed in GDS, provide an efficient reduced representation that captures large-scale structural rearrangements. Despite not being suited to the study of localized conformational changes, such as side-chain rotations, GDS can reveal details of the folding mechanism and associated time scales.

The remainder of this article is organized as follows. First, we apply the GDS workflow outlined above to a toy 2D protein folding landscape modeled as a rugged funnel. We confirm that one can define a suitable basis set for Markov model construction, without resorting to long MD trajectories beforehand; this basis is generated using intermediate configurations from a path-sampling algorithm defined on a discrete grid, in a similar fashion to our (more general) GDS algorithm. Second, we use our GDS/VAMP methodology to investigate folding of the designed GTT variant of the FIP35 WW domain,^30^ a single chain protein with 35 residues with a folding topology defined by two β hairpins. We find that using GDS—in conjunction with a physically motivated structure similarity metric defined in protein contact-map space—enables construction of a suitable basis set for a Markov model; we subsequently demonstrate the accuracy of this model by parametrization and kinetic evaluation using existing ANTON MD trajectory data.^6^ Importantly, this process definitively identifies a ‘kinetic trap’ within the folding pathway(s) of the FIP35 WW domain which previous conventional VAMP-based approaches struggled to resolve. As such, our results clearly highlight the potential of this combined GDS/VAMP approach to redefine exploration of protein folding dynamics, simultaneously offering a route to rapidly identifying elusive folding intermediates and kinetic bottlenecks that escape detection by more traditional partitioning methods.

## Methods

### Markov State Models and the Variational Approach

MSMs are discrete Markov chain models used to examine long-time dynamics of complex high-dimensional systems. Building an MSM starts with a partitioning (or discretization) of state space Ω, labeled {*A*_*i*_ ⊂ Ω}, from which a transition matrix **P** ^τ^ describing the transition probability between any of the two states, *i* and *j*, is defined as

1 $$P_{\mathrm{ij}}^{\tau }≔\mathrm{Pr}\left(x\left(\tau \right)\in A_{j}|x\left(0\right)\in A_{i}\right)$$

where **x**(t) = **x**_*t*_ ∈ Ω is the system configuration at time *t*. For the typical use-case of studying long time scale biomolecular dynamics for an *N*-atom system, **x**(*t*) represents a point in 3*N*-dimensional Cartesian space. The lagtime τ is chosen so that the system is approximately Markovian or “memoryless”, such that the state partitions {*A*_*i*_} are quasi-equilibrated across all nonrelevant “fast” modes (relative to τ).

Given that eq 1 is a linear model, we benefit from a range of results from spectral analysis. The transition matrix **P** ^τ^ can be decomposed via its eigenvalues and eigenvectors,

$$P^{\tau }\rho =\sum_{i}\lambda_{i}\left(\phi_{i}\cdot \rho \right)$$

where **ρ** is a vector describing probability density associated with each state-space partition (such that 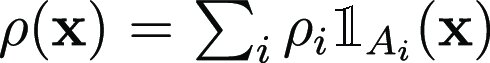), and (λ_*i*_, **ϕ**_*i*_) are eigenvalue/eigenvector pairs of the matrix **P** ^τ^ (in descending magnitude). The leading eigenvalue is always λ_0_ = 1 (corresponding to the stationary distribution), and the subdominant eigenvalues λ_1_, λ_2_,... characterize the time scales of the slowest relaxation processes in the system. These eigenvalues are related to relaxation times through *t*_*i*_ = −τ/ln λ_*i*_, and the associated eigenvectors describe the collective modes through which the system relaxes toward equilibrium. This spectral insight allows us to identify metastable states, construct coarse-grained few-state kinetic models, and understand dynamical bottlenecks through tractable transition path analysis within the system.^31–35^ A brief explanation of how the parameters of a MSM are fitted from MD trajectories is provided in the Supporting Information.

Selecting the partitioning of conformational space is integral to the quality of the MSMs. Early MSM approaches applied clustering algorithms to previously generated MD data to partition configuration space.^9,10,36^ The impact of both the clustering technique and the metric used to group kinetically connected configurations together has been the subject of several previous studies.^36–41^

In an effort to evaluate the choice of state-space partitioning, variational approaches were used to quantify and compare state-partition quality. The problem of building an accurate model of **P** ^τ^ can be reformulated as seeking a finite-rank approximation to the Perron-Frobenius (transfer) operator defined as

$$T^{\tau }\left[u\right]\left(x\right)=\frac{1}{\pi \left(x\right)}\int u\left(y\right)\mathrm{Pr}\left(y\to x,\tau \right)\pi \left(y\right)\mathrm{dy}$$

where (**x**, **y**) are system configurations, π­(**y**) is the stationary distribution, and *u*(**y**) is a state-space distribution weighted against the stationary distribution.^42^ A brief elaboration and demonstration of this operator’s properties is presented in the Supporting Information. More specifically, we demonstrate that a MSM is a finite-rank approximation to $T^{\tau }$ when using an indicator-function basis.

Importantly, the transfer operator $T^{\tau }$ obeys a variational principle analogous to that in operation for ground-state quantum chemistry. Instead of finding the lowest energy ground-state, however, here we aim to find eigenvectors that capture the slowest relaxation processes (see Supporting Information). In other words, we seek the eigenvector **ϕ** ^slowest^ such that

$$\phi^{\mathrm{slowest}}=\arg\max_{\phi }\frac{\left\langle \phi \right|T\left|\phi \right\rangle }{\left\langle \phi ,\phi \right\rangle }$$

offering a route toward assessing the validity and accuracy of a given state-space partition.^42^ However, the variational principle is not restricted to use of indicator functions as state-space basis functions, as is the case in traditional MSMs. Exploiting this variational principle is now a well-known framework for studying slow chemical processes, referred to as the variational approach to conformational dynamics (VAC).^13,42^

Estimating the transfer operator $T^{\tau }$ directly from MD data can lead to errors arising from off-equilibrium sampling effects. From a practical point-of-view, it is often preferable to instead estimate the closely related Koopman operator, $K^{\tau }$ , that propagates observables of a system in time, rather the probability densities. For an observable *f*, the Koopman operator is defined by

$$K^{\tau }\left[f\right]\left(x\right)=E\left[f\left(x_{t+\tau }\right)|x_{t}=x\right]=\int \mathrm{Pr}\left(x\to y,\tau \right)f\left(y\right)\mathrm{dy}$$

For a reversible system, the Koopman and transfer operator formalisms can be shown to be equivalent (see Supporting Information). The key strength of the Koopman operator, however, is that it makes no reference to the equilibrium distribution and is therefore more general. Furthermore, the Koopman operator (represented in some chosen basis) can be estimated directly from MD trajectory data—even if the data are short, sparse, or out of equilibrium—making it significantly more practical for data-driven methods.^43^ As for the transfer operator, a variational principle applies for Koopman operators, referred to as the “Variational Approach to Markov Processes” (VAMP).^14,42,44^

One particularly successful and popular approach to address this problem is the use of VAMPNets. This approach seeks neural-network-parametrized basis functions, χ_*i*_^*NN*^ that maximize the variational score, improving state-space resolution and kinetic accuracy.^15^ Whether an MSM or a VAMPNet is used, the problem can be categorized as (A) constructing an effective and accurate state-space partitioning or basis, and (B) estimating the elements of the transition matrix *P*_*ij*_^τ^, transfer operator $T_{\mathrm{ij}}^{\tau }$ or the Koopman operator $K_{\mathrm{ij}}^{\tau }$ from a given collection of MD trajectories. As noted, (A) has been the subject of many studies employing clustering or optimizing basis functions from previously generated MD data; this in essence couples the choice the of basis functions with the expensive generation of MD data for problem (B). The coupling introduces an undesirable trade-off; if one were to choose too large of an initial basis set (either discrete or continuous) in an attempt to accurately resolve configurational space, then it may be too difficult to generate a sufficiently large MD data set for statistical computation of transition probabilities. On the other hand, basis sets that do not capture essential conformations and associated kinetics could overlook important slow transitions, metastable states, and kinetic traps.

Existing work now attempts to tackle the issue of requiring initial large MD data sets by iteratively building a Koopman model with some existing MD data, subsequently using the model to inform further MD simulations in areas of poor sampling.^16,17^ While less demanding that converged MD simulations of slow systems, this iterative approach still represents a computational-time barrier.^16^ Alternatively, there has been work on estimating parameters for an MSM (i.e., using discrete state partitions) without the need for any MD simulations,^45–47^ but such efforts are restricted to either specific simple partitions that suffer the curse-of-dimensionality, or Voronoi partitions which are nontrivial to implement for complex systems like proteins.

Based on this discussion, one would ideally seek an approach to identify minimal *important* regions of configuration space, before calculating the relevant statistical properties for MSM construction. The approach discussed below addresses this challenge.

### Graph Driven Sampling

In this article, we propose to replace MD simulations for configuration space partitioning with an alternative, order-parameter-free scheme that enables fast, accurate and physically interpretative sampling of relevant protein-folding configurations. We have recently shown how the ensemble of physically realizable protein-folding pathways can be generated using directed walks in the space of inter-residue contact-maps. Combined with a back-transformation scheme to move from protein contact-maps to Cartesian coordinates, we have demonstrated how this approach can generate protein-folding ensembles without resorting to extensive MD simulations.^22,23^ Here, we propose that this GDS scheme can be used to create a compact and accurate basis for Koopman model construction, circumventing the need for MD simulations or order-parameter-based partitioning, and therefore providing a significant advantage for targeted simulation strategies.

In the following, for an *N*-residue protein, the contact-map **G** is an *N* × *N* square matrix that defines whether or not two residues in the protein structure are in close-contact according to a predefined distance criteria:

2 $$G_{\mathrm{ij}}=\{\begin{array}{ll} 1, & \mathrm{if}\;\left(r_{\mathrm{ij}}<r^{c}\right)\\ 0, & \mathrm{otherwise} \end{array}$$

Here, *r*_*ij*_ is the distance between residues (*i*, *j*) (defined using the coordinates of their respective C_α_ carbon atoms) and *r* ^c^ is an appropriately chosen cutoff distance (typically 8 Å, but a range of cutoff distances up to 11 Å have shown to be similarly sufficient^48^). This representation offers several advantages. Most importantly, because contact-maps encode secondary and tertiary structure information while remaining invariant to rotations and translations, they provide a compact and physically meaningful description of the folding process, as exploited in previous work.^49,50^ Furthermore, employing this discretization, a protein-folding pathway can be represented as a sequence of ‘hops’ between contact-maps, where each consecutive hop represents an elementary conformational transition; we refer to this path representation as a graph sequence, [**G** ^0^, **G** ^1^,..., **G** ^*N*^], where **G** ^0^ is an “unfolded” contact-map (such that *G*_*ij*_ = 0 ∀ *i*, *j*) and **G** ^*N*^ is the folded state.

Here, we briefly recap our initial strategy to identify graph sequences that—when back-transformed into a corresponding Cartesian-coordinate representation—propose physically viable folding pathways. We start with an initial (typically random) sequence of graphs [**G** ^0^, **G** ^1^,..., **G** ^*N*^], and an objective function *F* assessing path connectivity and continuity. Following our previous study, we use:

$$F=\sum_{i=1}^{N-1}\mathrm{f̅}\left(G_{i}\right)$$

where the contact-map-dependent function *f̅*(**G**_*i*_) is

3 $$\mathrm{f̅}\left(G_{i}\right)=H\left(f_{\mathrm{SCH}}\left(G_{i},G_{i-1}\right)-k_{c}\right)+H\left(f_{\mathrm{SCH}}\left(G_{i},G_{i+1}\right)-k_{c}\right)+\left|f_{\mathrm{SCH}}\left(G_{i},G_{i-1}\right)-f_{\mathrm{SCH}}\left(G_{i},G_{i+1}\right)\right|$$

Here, *H* is the Heaviside step function, *k*_c_ is a parameter that seeks to enforce path continuity in contact-map space (with *k*_c_ = 0.15, following our previous studies), and *f* ^_SCH_^ is the shortest contact hop (SCH) metric introduced in our previous study as

4 $$f_{\mathrm{SCH}}\left(G^{A},G^{B}\right)=\frac{1}{N^{2}}\sum_{i,j}^{N}\frac{\left|S_{\mathrm{ij}}^{A}-S_{\mathrm{ij}}^{B}\right|}{\max\left(S_{\mathrm{ij}}^{A},S_{\mathrm{ij}}^{B}\right)}$$

Here, *S*_*ij*_^*A*^ is the shortest-distance between two residues (*i*, *j*) as calculated using Seidel’s algorithm for contact-map **G** ^*A*^. Importantly, we note that the SCH metric only requires contact-map information—not real-space Cartesian coordinates. As such, it is fast to evaluate, contributing to the overall efficiency of GDS. Equation 4 is a key component of our approach: we have have previously demonstrated that the SCH metric captures structural similarity between different protein contact-maps more accurately than the more commonly used Hamming distance,^23^ a feature which ultimately leads to better MSM resolution, as shown later. Discrete optimization of the folding-path sequence under the influence of *F* seeks to steer the search away from violation of connectivity criteria; the third term in eq 4 seeks to ensure that an intermediate contact-map **G**_*i*_ is approximately equidistant between neighboring contact maps (*i* + 1) and (*i* – 1), while the first two terms punish significant discontinuities in adjacent contact-maps.

Starting from an initial contact-map sequence, the GDS algorithm performs Markov chain Monte Carlo updates using *F* as an objective function for Metropolis-Hastings updates. The update “moves” involve randomly selecting a contact-map **G**_*i*_ (*i* ≠ (0, *N*)) and performing a small random number of “bit flips” (which perturb *G*_*ij*_ ∈ [0,1]). A linearly decreasing simulated annealing (SA) temperature schedule is used to encourage initial path searching before refining *F* in later iterations. Given that every contact map generated in this procedure is not necessarily physically realizable (due to, for example, violations of the triangle inequality), a reconstruction of the Cartesian coordinates from the contact map is attempted after each bit flip; further details of this back-transformation are given in our previous work.^23^ Overall, the GDS path-generation algorithm is very fast; each path typically requiring less than a minute of calculation time on a standard laptop.

In protein folding, slow dynamic motions are often directly related to the formation of native contacts and the organization of key secondary structures. In previous work, we used energy barriers computed using nudged elastic band (NEB) calculations on coarse-grained interaction models to select a small set of “likely” folding pathways. We further clustered this set of pathways using the Frechét metric^51^ to identify common motifs among pathways. In principle, however, there is a great deal of freedom in selecting pathways of interest during a postprocessing procedure. As noted above, the variational approach to decomposing dynamical systems can yield direct insight into the slow modes and relaxation time scales governing the kinetics. Therefore, we expect a basis informed with intermediates extracted from GDS simulations to serve well in decomposing our dynamical system under the VAMP framework; this is the key idea of this article.

### Markov Models in Contact-Map Space

Many computational studies of protein folding have made use of the assumption that the formation of native contacts categorizes the folding mechanism.^52,53^ However, simply using the fraction of native contacts, *Q*, as a reaction coordinate for folding has been shown to have severe non-Markovian artifacts.^54–56^ It also lacks structure specificity; a particular *Q* can be obtained from several topologically distinct conformations, such that this approach may struggle to resolve multiple pathways or trapped intermediates. It has been shown that—by finding optimal weightings for different contacts in a similar reaction coordinate to *Q*—one can better interpret the resulting modified reaction coordinate and gain insight into critical events within the folding path.^57^ However, the same work also found that the highly cooperative nature of contacts lead to a degeneracy over the optimal reaction coordinates. This finding is not surprising in the context of our discussion on the choice of metrics to measure structural similarity of contact maps,^23^ where the influence of a specific contact on the structural similarity of protein structure can vary, and depends on the environment described by the rest of the topology.^40^ Instead, a Markov model constructed with state definitions that can both minimally cover *and* successfully distinguish the relevant parts of state space would be anticipated to be expressive enough to identify different pathways and intermediates.

Given that our previously reported SCH metric (eq 4) is able to effectively capture structurally similarity, we argue that using SCH-based radial Gaussian basis functions centered around significant intermediates (i.e., contact-maps) should serve as a “good” set of basis functions for construction of a Markov model describing protein folding. Indeed, contact-maps serve as a compact descriptor with great specificity for protein-folding topologies, and we have shown already that the SCH metric is effective in identifying collections of correlated structures during folding.^23^ In turn, this approach offer a good balance between dealing with the high-dimensional fully atomistic description of a protein and the often-too-simplified native contacts coordinate *Q* that can fail to capture important complexities of the folding process.

The GDS framework presented above is an ideal approach for generating intermediate folding structures {*G*_*i*_} to define a set of basis functions evaluated on the space of contact maps. In other words, we propose here to choose the basis {χ_*j*_} for our Koopman expansion to be

$$\chi_{j}\left(r\right)=\exp\left(-\frac{f_{\mathrm{SCH}}{\left(G_{j},G\left(r\right)\right)}^{2}}{2\gamma^{2}}\right)$$ 5

where the *f*_SCH_ is given by eq 4 and γ is chosen depending on the required resolution for the problem. These basis functions are readily and efficiently generated by GDS without the need for large numbers of potential energy surface (PES) evaluations or long time scale MD trajectories, and can be subject to several postprocessing steps such as thermodynamic or kinetic filtering.^22,23,58–60^ Furthermore, the enforced connectivity of paths guarantees sufficient coverage to compute transition-rate statistics starting from the unfolded to folded state of the protein, as we demonstrate below. Most importantly, by construction, the GDS-generated paths would be expected to account for the small—yet important—fraction of state-space that is directly relevant to the folding mechanism. Thus, the GDS framework should provide a significant sampling advantage over randomly selected basis functions, while constructing a localized basis set using GDS represents a more feasible computational task than constructing a basis set spanning *all* state-space.

To summarize, the overall workflow for kinetic model construction proposed here is shown in Figure 1, alongside a representation of the “standard” MD-based workflow. As shown in the next section, we find that the GDS-based route to kinetic model construction more accurately captures folding time-scales (in the variational sense) compared to previous MD-based studies, yet provides an intuitive and physically interpretable model for complex protein-folding processes.

**Figure 1:**
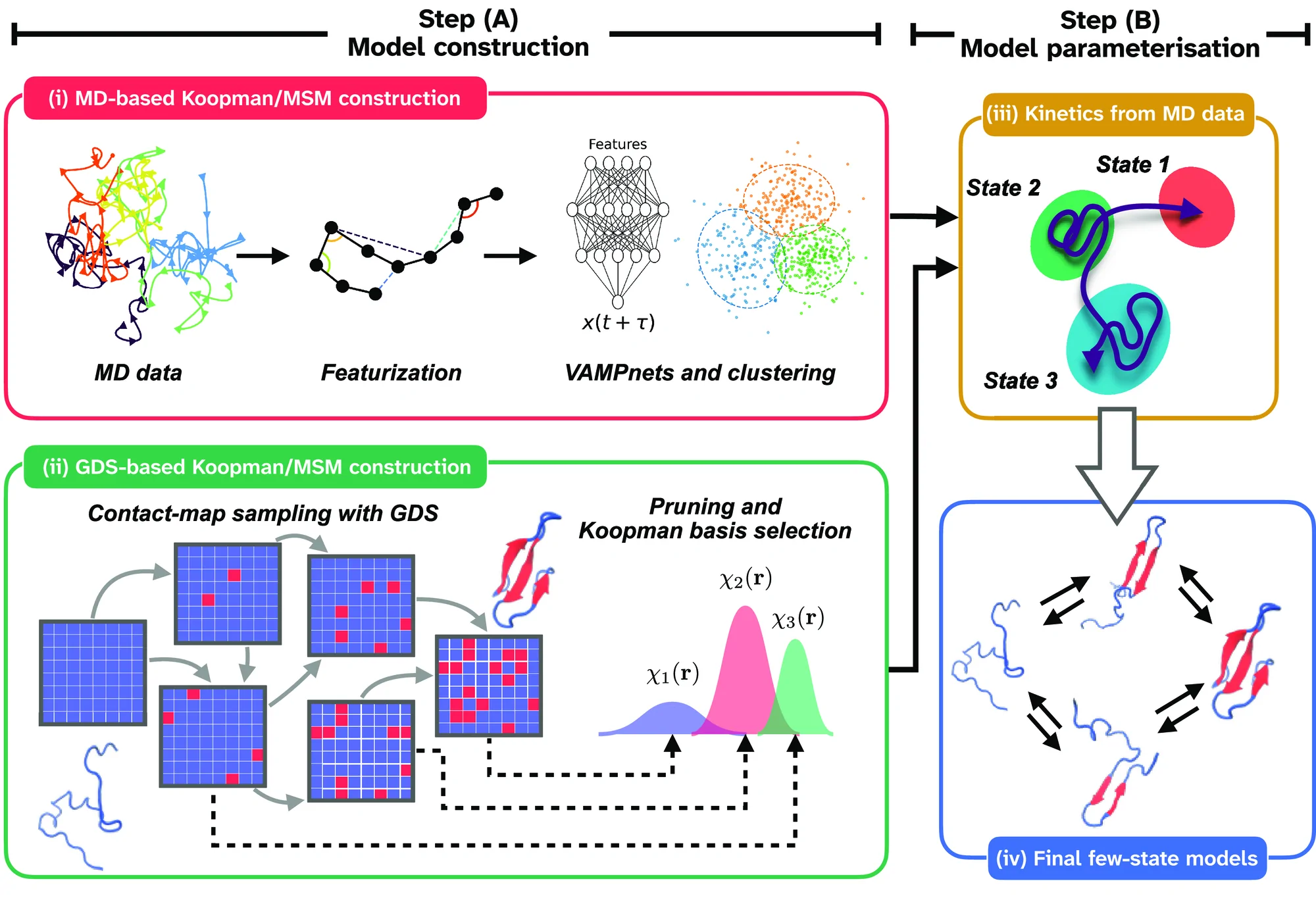
MSM construction demands both (A) model construction and (B) parametrization. (i) outlines the common MD-based strategy, in which MD trajectory data is used as input for structure featurization and VAMPnet basis-learning. In contrast, model-construction pathway (ii) proposed in this work employs direct sampling in contact-map space—along with a physically intuitive basis- to provide a reduced (yet accurate) kinetic model of protein folding. In both MSM construction pathways considered here, kinetic parametrization (iii) is obtained from existing MD trajectory data, enable direct comparison of different (iv) few-state models on an equal footing.

## Results and Discussion

To demonstrate how GDS can be used to construct an accurate MSM for complex dynamic processes, we begin here by presenting a illustrative example for a model two-dimensional (2D) “folding funnel” representative of the rugged landscapes traversed by more realistic protein-folding paths. Following this proof-of-concept, we turn to construction of a Markov model for a much more realistic protein-folding process, namely folding of the 35-residue WW domain protein (described by an atomistic force-field with explicit water). We demonstrate that GDS constructs a basis set for Markov modeling that is accurate, interpretable, and capable of resolving features—such as important kinetic traps—that have proven difficult to sufficiently resolve using previous partitioning approaches. Furthermore, we subsequently demonstrate that the time scales and mechanism of folding extracted from our GDS-based MSM agree with (and, indeed, improve upon) previous estimates.

### A Rugged 2D Protein-Folding Landscape

We first illustrate our GDS-based approach to construct a Markov model for a 2D energy landscape that is qualitatively reminiscent of the “rugged funnels” associated with protein folding.^61^ The model PES used here is defined as

6 $$V\left(x,y\right)=A\left(x^{2}+y^{2}\right)+Ce^{-\alpha \left(x^{2}+y^{2}\right)}+Se^{-k{\left(\sqrt{x^{2}+y^{2}}-r_{0}\right)}^{2}}+\sum_{i=1}^{N}D_{i}e^{-\beta_{i}\left[{\left(x-x_{i}\right)}^{2}+{\left(y-y_{i}\right)}^{2}\right]}$$

The model is expressed in arbitrary reduced units of *t*_0_ (time), *l*_0_ (length), and *m*_0_ (mass). The first term, with *A* = 1, defines a harmonic funnel centered at the origin, representing the overall thermodynamic bias toward the folded (native) state. The second term, a Gaussian well, with *C* = −30 and α = 3, introduces a deep, narrow minimum at the origin, mimicking the energetic stabilization of the native structure. An additional Gaussian ridge term, centered at radius *r*_0_ = 2 with inverse-width *k* = 2 and height *S* = 30 creates a circular barrier around the native basin, emulating an entropic bottleneck.

To model the rugged nature of real protein-folding surfaces, we superimpose *N* = 50 randomly positioned local perturbations. Each perturbation is modeled as a Gaussian centered at (*x*_*i*_, *y*_*i*_) ∼ *U*[−3, 3]^2^, with depth *D*_*i*_ ∼ *U*[−20, 5] and inverse-width β_*i*_ ∼ *U*[1, 3]. These randomly distributed traps are designed to resemble metastable intermediate or misfolded states—local basins into which the system can become transiently trapped for long periods of time. They also create low or barrier-less routes for the system to bypass the entropic bottleneck term.

As illustrated in Figure 2, the typical landscapes generated using eq 6 are comparable to reduced-dimensional representations of real protein folding PESs, where the energy surface is typically dominated by a few low-lying minima separated by high barriers and several narrow transition paths. Thus, it captures the key dynamical challenge in realistic systems, namely investigation and characterization of rare-event transitions and long-lived traps within a rugged energy landscape.

**Figure 2:**
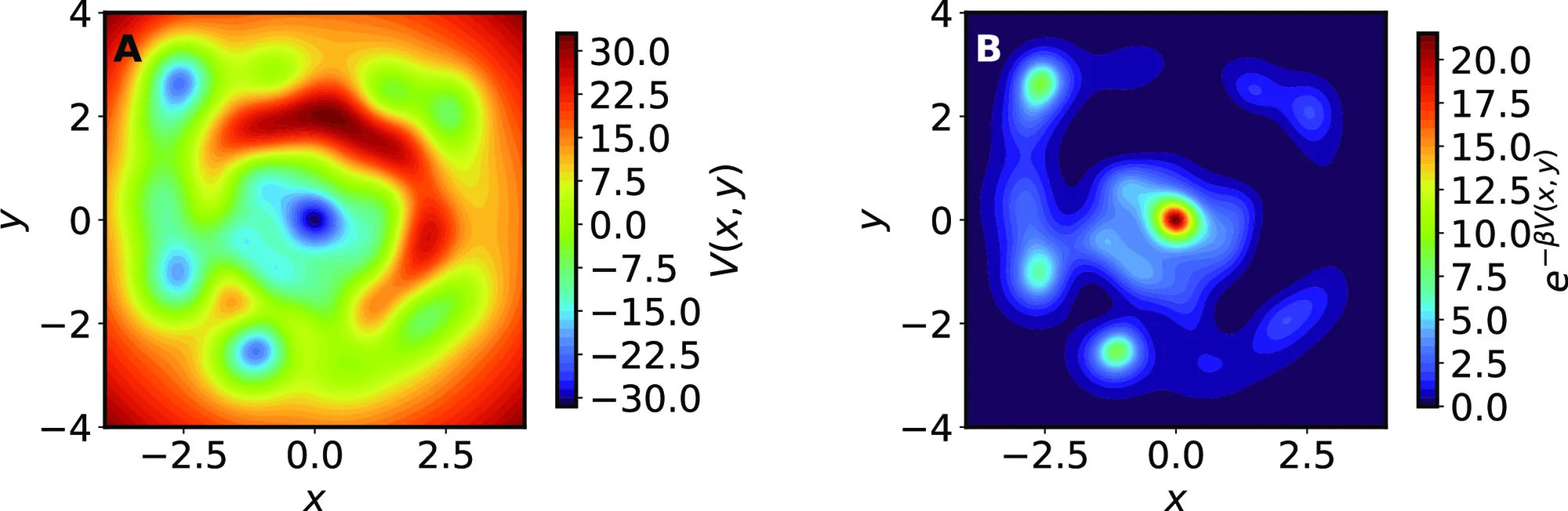
Contour plots of of the 2D PES *V*(*x*, *y*) (see eq 6) and the corresponding Boltzmann distribution for β = 0.1. The left panel (A), a contour plot of the PES, has features reminiscent of low-dimensional projections of protein folding landscapes, which are typically dominated by a few low-lying minima separated by high barriers and several narrow transition paths. The right panel (B), shows a contour plot of the Boltzmann distribution with inverse temperature β = 0.1. The distribution is heavily dominated by the folded conformation −*x*, *y* = (0, 0).

To generate an ensemble of folding paths that are discretized in a similar fashion to the GDS scheme, we first define a discrete grid on the 2D PES. We note that, for general protein systems as studied below, the residue–residue contact map offers an ideal motivated discretization. However, in this 2D model case, a simple square grid tessellating space serves as a sufficient discretization. The square grid centers, **C**_*i*_, were chosen to be 11 × 11 points equally displaced across [−4, 4]^2^, as shown in Panel C of Figure 3.

**Figure 3:**
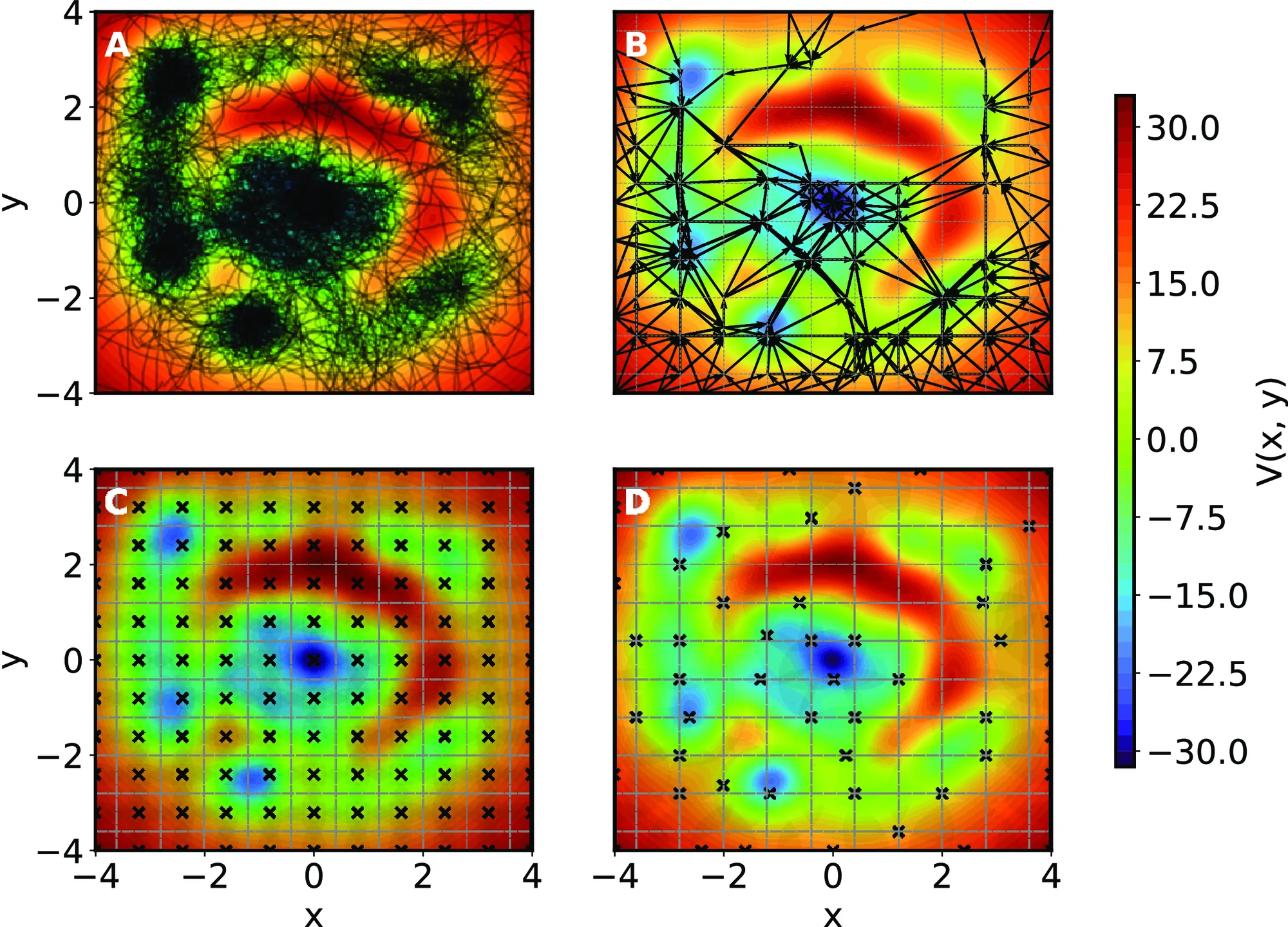
GDS exploration of pathways on a rugged 2D folding funnel. Panel (A) shows 100 Langevin trajectories of length 50 time-units each. Panel (B) presents the 200 GDS paths used to build a basis for a Markov model. Panels (C) and (D) present the locations of the basis centers for Markov model construction; the centers are marked with a cross, and a faint circle of radius 1 illustrates space coverage. Panel (C) presents the 121 centers of a uniform space-covering basis. Panel (D) shows the sparse basis generated from GDS simulations.

An initial guess for a sequence of squares (i.e., configurations) to visit is made with *C*_0_ = *C*_1_ = ... = *C*_*N*–1_ = (*x*, *y*)_boundary_ where (*x*, *y*)_boundary_ is a uniformly sampled point on the boundary. The final square *C*_*N*_ = (0, 0) represents the folded state. Just as in our GDS framework, we perturb a randomly selected *C*_*i*_ with a move to an adjacent cell and accept or reject the move with a Metropolis-Hastings criteria based on the simplified objective function, *f̃* defined as

$$\mathrm{f̃}\left(C_{i}\right)=H\left({∥C_{i}-C_{i-1}∥}_{1}-k_{\mathrm{cont}}\right)+H\left({∥C_{i}-C_{i+1}∥}_{1}-k_{\mathrm{cont}}\right)$$

We note that this objective function is comparable to that employed in GDS simulations in seeking to generate directed walks through configuration space that end at the folded state. In total, 10^3^ sequences of length *N* = 20 were generated by minimization of this objective function (with *k*_cont_ = 1 in all cases). To generate each path, 10^5^ SA iterations were performed, with the temperature linearly cooled from *T* = 10 to *T* = 0.1. Once this procedure was completed, each sequence was “back-mapped” into a piecewise-linear path in **R** ^2^; the “back-mapping” consists of linearly interpolating the minima of each of the visited square. Finally, we selected the 200 paths with lowest total uphill energy change as representative examples of folding trajectories with which to construct a Markov model partitioning.

To generate a point of comparison, we also used classical MD trajectories to sample the 2D configuration space of the folding landscape. Using classical MD simulations to sample trajectories can be expensive from a time scale point view, because the system can spend a large fraction of time trapped in metastable states. For the 2D potential studied here, we initiated 1000 Langevin trajectories, each for a total time *t* = 50 time-units, starting from (*x*, *y*) ∼ *U*[−4, 4]^2^. All trajectories were simulated with a Boltzmann factor β = 0.1, a damping coefficient of 1 (mass/time)-units, and a time-step of 0.001 time-units. As shown in Figure 3, this provides sufficient dynamical sampling of the configuration space, but the vast majority of the exploration lies trapped within the metastable areas (as expected based on temperature and landscape characteristics).

MD data sets like that illustrated in Figure 3A would normally be used to identify clusters and state definitions before calculating and parametrizing a corresponding MSM. Instead, to illustrate the utility of our approach, we first select a basis set derived from discrete paths generated using GDS; furthermore, we show that this approach to generating a basis does not undermine accuracy. We choose to build a “ground truth” model using a basis that systematically covers the whole space of interest; specifically, we use a Gaussian basis centered around the 11 × 11 = 121 points uniformly distributed across the domain [−4, 4]^2^. In contrast, to select a suitable basis using GDS, we selected intermediates from the GDS discrete paths, and pruned centers that were within a distance *d* = 0.8. This approach ultimately selected 50 Gaussian centers that were distributed over the expected regions of interest. We note that—with increasing dimensionality—the ratio between the number of centers required to cover *all* configuration space (rather than transition-path-associated space) is expected to grow dramatically.

Before building a Markov model, we selected the lagtime τ. Typically, τ is chosen to capture slow processes and equilibrate fast processes; in other words, the lagtime τ should be selected so that the system behaves in a Markovian fashion. However, no reliable method currently exists to achieve this *a priori*. We use a simple heuristic discussed in the Supporting Information, from which we find that 1.5 time-units was a suitable decision.

Following selection of lagtime τ, we fit a reversible Koopman model using the unbiased Koopman reweighting algorithm.^44^ We then compared the two models—specifically the model generated using GDS basis selection and the model generated using a grid basis—by evaluating the eigenvalues of the three slowest modes (which, according to the variational principle, should be maximized). We find that the model built with the GDS basis yields eigenvalues of (0.748, 0.670, 0.590), in excellent agreement with those found with using the complete space-covering basis (0.750, 0.672, 0.594). For a more detailed validation, we compared the three corresponding eigenfunctions of both models (Figure 4). Again, these are in excellent agreement with each other; most importantly, the characteristic dynamics associated with each reaction-coordinate (RC) are clearly aligned. For example, for the slowest RC (Figure 4A1 and B1), there is a clear “diagonal bias” in both eigenmodes, indicating that the slowest RC reflects the presence of the inner PES barrier to the folded-state (Figure 3) and the lowered energetic barriers in the lower left-hand corner of the PES (Figure 2).

**Figure 4:**
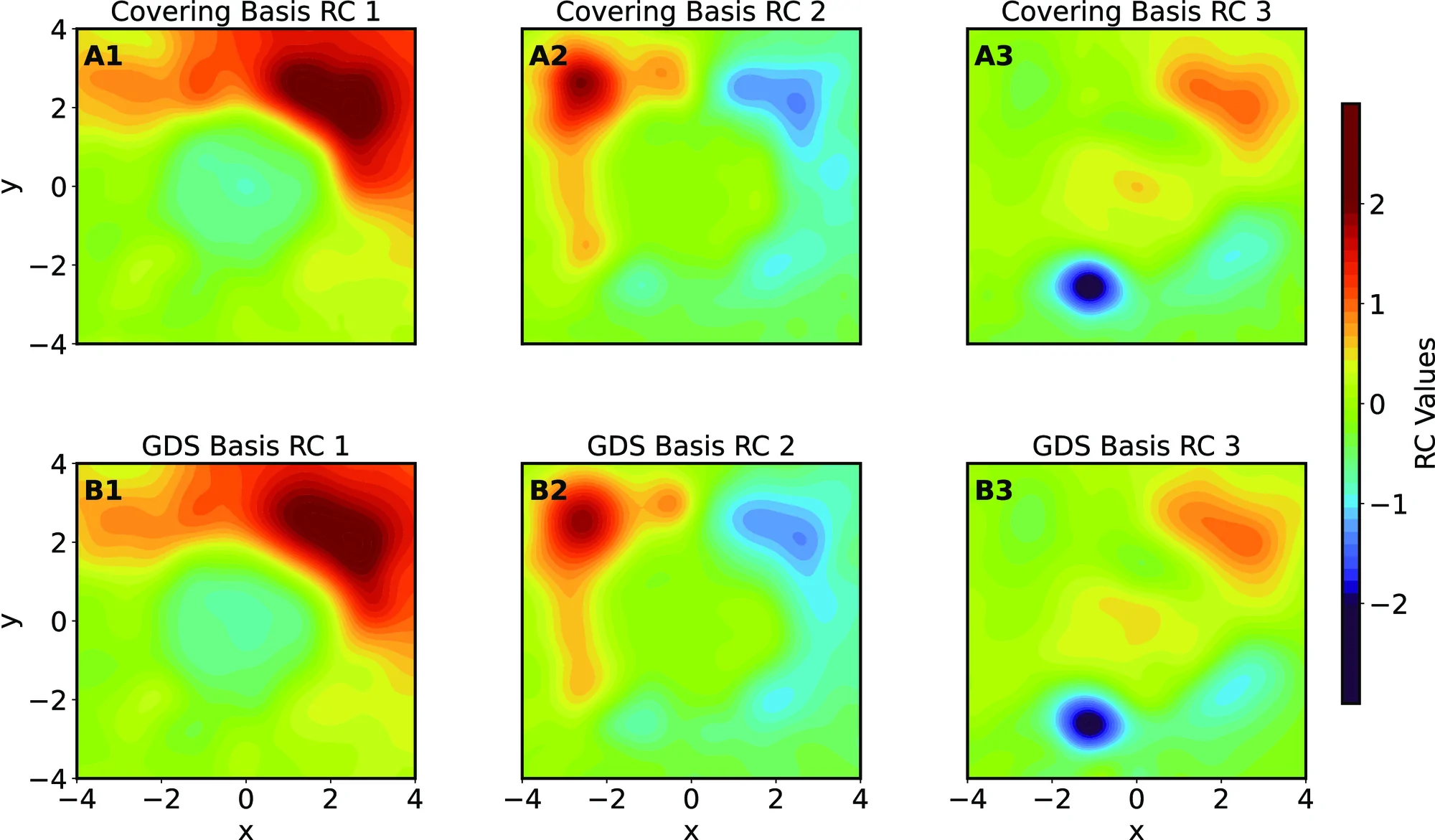
Comparison of contour plots representing the three slowest reaction coordinates (RCs) expanded in the complete-space-covering basis (Panels A1–A3) or the GDS basis (Panels B1–B3). We observe excellent agreement between RCs obtained by the two different approaches, despite using a much smaller basis set in the GDS case.

To summarize, the results presented here for a rugged 2D model of a protein-folding landscape demonstrate that the GDS framework can be used to generate a compact and accurate basis for Markov model construction. In other words, we have shown that efficient GDS simulations can—in principle—replace extensive MD trajectory sampling in the search for a Markov basis. However, the broader utility of GDS in this context must clearly be demonstrated for a more realistic, higher-dimensional problem. In the following section, we demonstrate that the success of GDS for model construction readily carries over to constructing accurate Markov models for a fully atomistic model of a folding protein in a solvent environment.

### GDS-Constructed Markov Model for WW Domain Folding

To demonstrate the wider applicability of our GDS methodology in the context of an actual protein-folding problem, we investigated the folding of the GTT variant of the FIP35 WW Domain (Figure 5a). This 35-residue protein represents a prototypical folding example, being one of 12 “fast folders” that have been studied by long-time scale MD simulations using the dedicated ANTON computer.^6^ The available MD data for this protein comprises two MD trajectories totalling a simulation time of more than 1 ms. The folded state was chosen as a configuration with a high value of the *Q*_DESRES_ parameter constructed in the initial ANTON study; this order parameter measures structural similarity to the known folded state.^6^

**Figure 5:**
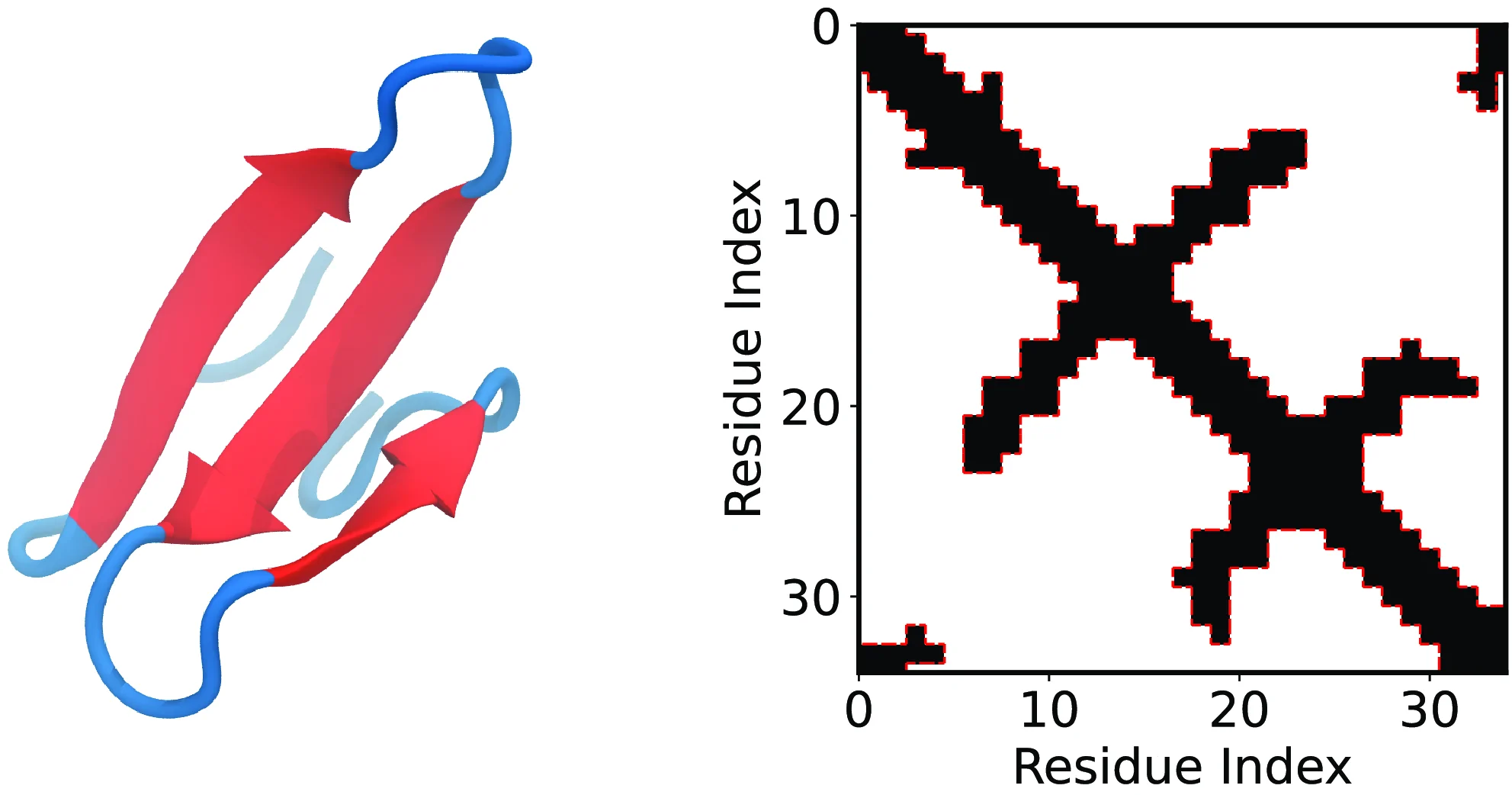
Folded state of the FIP35 WW domain protein (left), with β-sheet secondary structures highlighted in red. On the right, the corresponding contact map of the folded WW domain is shown, where black pixels indicate a contact between two residues (eq 2). Arbitrarily, we also define the diagonal elements as *G*_*ii*_ = 1. As expected, two perpendicular regions are seen in the native-state contact-map of the FIP35 WW domain, corresponding to interactions between the β-sheets.

To proceed, we initiated 100 GDS runs starting from a “null” contact-map representing an entirely unfolded configuration of the protein sequence; the contact-map of the folded protein was used as the target end-point for all GDS trajectories. The GDS graph sequences were chosen to be of length *N* = 10, inclusive of unfolded and folded end points, and the target objective function of eq 3 used the parameter *k*_cont_ = 0.05. This choice was made based on Figure S2 in the Supporting Information (SI), where it is clear that structures within this distance are kinetically accessible. Our SA procedure employed 10^4^ iterations of single-contact perturbation proposal moves, with a Metropolis-Hasting acceptance-rejection scheme starting with a temperature of 10^–3^ decreasing linearly (we note that the temperature scale does not map on to typical physical temperatures because the units and scale of the objective function in eq 3 are somewhat arbitrary; instead, the initial temperature used here was chosen using initial test simulations). Each single-contact perturbation proposal involves randomly selecting a graph from the current sequence, selecting a random contact for “bit-flipping” (breaking or forming a contact), then proceeding to attempt to reconstruct the proposed graph.

The reconstruction is key here—otherwise, the GDS optimization encounters a large number of nonphysical graphs with distance restraints that cannot be satisfied in 3D space.^23^ Reconstruction involves the minimization (using LBFGS^62^) of a simple polymer-chain empirical PES combined with a so-called Graph Restraining Potential (GRP):

7 Vrecon(rα,G)=Vpoly(rα)+VGRP(rα,G)=∑i=2Nrkpoly(|riα−ri−1α|−σ)2+VGRP(rα,G)

Here, **r**_α_ refers to the positions of the *C*_α_ atoms and *V*_poly_ is a polymer-chain PES comprising harmonic bonding terms with *k* = 115.6 kJ mol^–1^ nm^–2^ and σ = 0.38 nm (the typical distance between neighboring *C*_α_). The artificial PES *V*_GRP_ is a function of both coordinates and a specific contact map that is the target for reconstruction. Given a contact map, **G** = (*G*_*ij*_) (defined according to eq 2 with a cutoff distance of 8 Å), we use the following distance restraint potential on *C*_α_ atomic positions:

$$V_{\mathrm{GRP}}\left(r_{\mathrm{ij}}^{\alpha },G_{\mathrm{ij}}\right)=\{\begin{array}{ll} k_{\mathrm{GRP}}{\left(r_{\mathrm{ij}}^{\alpha }-r^{\mathrm{con}}\right)}^{2}, & G_{\mathrm{ij}}=1,r_{\mathrm{ij}}^{\alpha }>r^{\mathrm{con}};\\ k_{\mathrm{GRP}}{\left(r_{\mathrm{ij}}^{\alpha }-r^{\mathrm{steric}}\right)}^{2}, & G_{\mathrm{ij}}=1,r_{\mathrm{ij}}^{\alpha }<r^{\mathrm{steric}};\\ k_{\mathrm{GRP}}{\left(r_{\mathrm{ij}}^{\alpha }-r^{\mathrm{con}}\right)}^{2}, & G_{\mathrm{ij}}=0,r_{\mathrm{ij}}^{\alpha }<r^{\mathrm{con}};\\ 0, & \mathrm{otherwise} \end{array}$$

where *k*_GRP_ = 1000 kJ mol^–1^ nm^–2^, *r* ^steric^ = 2 Å and *r* ^con^ = 8 Å. Previous work has shown that minimization of the GRP can lead to contact maps that are “close” to the target contact map when measured with the SCH metric. As a result, when each GDS run is complete, a pathway of intermediate “physical” contact maps from unfolded to folded graphs is produced. Overall, the GDS algorithm for generating folding paths and intermediate structures is very fast; for example, each path-generation simulations requires less than 5 min run-time on a standard laptop.

The resulting contact-maps obtained from the GDS procedure sample possible physical pathways of intermediate structures connecting the unfolded and folded states. However, given the simple nature of eq 7, the sampled contact-maps might correspond to structures that are not stable on the relevant atomistic PES. To address this, we back-transformed from contact maps to fully solvated protein structures in Cartesian space, and subsequently allowed these structures to relax on the PES using short MD runs. This process biases the generated path-ensemble toward sampling more stable intermediates on the folding landscape. Here, intermediate contact maps were ”back-mapped” into an all-atom, explicit solvent system modeled via the GROMACS MD package^63^ using the CHARMM36m-TIP3P force-field.^64,65^ In this “back-mapping” procedure, we first obtain a biased structures in the fully atomistic protein–water system that is consistent with the current target contact map. This is achieved by running a high temperature MD simulation with a strong *V*_GRP_ bias applied, resulting in quickly decorrelated configurations that respect the GRP restraints. We subsequently cool this configuration (again, still applying *V*_GRP_ bias) to obtain a more thermodynamically stable representative structure. Finally, we remove *V*_GRP_ restraints and perform a short MD simulation in which the representative solvated protein structure relaxes further (noting that the contact-map is now free to “drift” from the original target). Notably, this simple procedure only requires around 1 ns of MD simulation time, which itself only requires a few minutes of ‘real-world’ time. Further details of this procedure are given in the SI. In total, relaxing the 800 GDS intermediates requires approximately 800 ns of aggregate MD. Importantly, these simulations are not used to construct the basis itself, which is already generated by GDS, but only to obtain locally relaxed atomistic representations. In comparison, Kleiman and Shukla used approximately 1, μs of aggregate MD in an iterative VAMPbased adaptive sampling scheme for a similarly sized fast-folding protein.^16^ Despite showing significant improvements over previous schemes, Kleiman and Shukla’s scheme was not able to explore more than small conformational changes from the folded structure. The goal in these schemes is similar to our goal here: constructing a basis such that MD can be sampled efficiently. However, Kleiman and Shukla’s results highlights both the key distinction and advantage of our GDS scheme has over the iterative approaches; iterative approaches still require non-negligible MD simulations to progressively learn the relevant configurational representation, whereas GDS provides a suitable basis *a priori* with negligible MD costs.

In total, GDS sampling yielded 800 physically relevant intermediate contact-maps; this number was further pruned to 608 by removing closely similar structures (specifically, a structure was pruned if it had a SCH distance metric value of less than 0.1 to any other existing structure). Finally, the overall Koopman model basis set was doubled in size to 1216 basis functions by placing two basis functions (eq 5) at each retained contact-map; these basis functions used different γ-values of 0.1 and 0.2, respectively. While not essential, we find that using this approach ensures better coverage of configuration space and a more accurate Koopman model.

We note that GDS does not rely on time scale separation; instead, GDS generates nonbiased folding pathways that—by construction—connect unfolded and target folded structures through pathways in “contact-map space”. The concepts of rates and time-scales emerge after GDS construction of a MSM basis; here, we obtain this kinetic data using available ANTON MD trajectories as a proxy, enabling model comparison against previous work. GDS does not guarantee sampling over slow dynamic modes, but does (by construction) generate paths that definitively fold from an unfolded structure to the final target structure. We expect that such paths would *a priori* contain intermediate structures associated with the slowest dynamic modes.

As noted above, fitting the Koopman operator requires selection of an appropriate lagtime over which the “fast” degrees-of-freedom in the system effectively equilibrate. We perform a similar heuristic analysis to that of the 2D model potential above, which is presented in the Supporting Information. We find that a lagtime of τ = 100 ns was an appropriate choice. A Koopman operator represented in the GDS basis was subsequently fit with that lagtime. For this purpose, we used the Koopman reweighting algorithm implemented in the deeptime package.^66^ Here, we generated a Koopman model describing the three slowest relaxing modes of interest. The resulting time scales were 10.6, 0.6, and 0.2 μs, computed by $t_{i}=-\frac{\tau }{\ln\left(\lambda \right)}$ , where τ is the lagtime. The projections of the MD data along these three slowest Koopman modes are shown in Figure 6.

**Figure 6:**
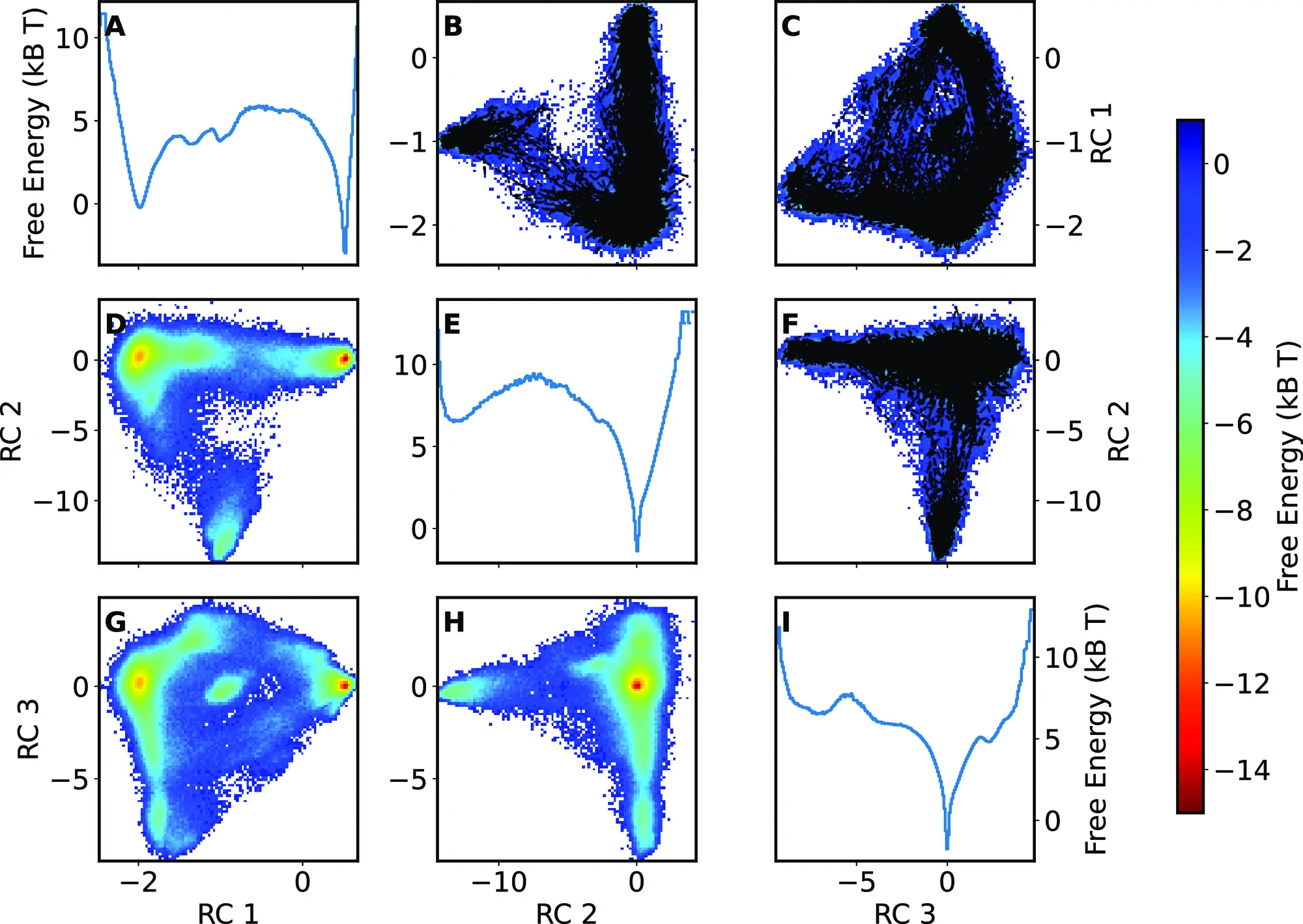
Free energy projections of the WW domain protein along the three slowest modes, RC1–C3. Along the diagonal, panels {A, E, I}, are the free energy curves along the separate reaction coordinates. The off-diagonal panels show 2D Free energy surfaces projected along two of the modes. Panels D, G, and H are the free energy surface plots for RC2 against RC1, RC3 against RC1, and RC3 against RC2, respectively. The upper triangular off-diagonal panels B, C, and F are reflections of panels D, G, and H, respectively, but with samples of the MD trajectory data overlaid. As further examined in Figure 7, we see that RC1 (the slowest mode) corresponds to the folding coordinate. RC2 distinguishes a stable kinetic trap that must unfold to enable progress to the native folded structure.

The slowest reaction coordinate (RC), corresponding to the folded-unfolded transition, correlates well with findings from previous studies.^18,67,68^ In Figure 6A, the free energy calculated along RC1 reveals two deep minima, simply corresponding to the folded and unfolded states; diagonal plots along RC2 and RC3 confirm that these RCs correspond to motion in the direction of specific higher-energy minima of the free-energy surface. However, the two-dimensional plots of RC pairs reveal further underlying structure to the free-energy surface. For example, plotting the free energy in the space spanned by (RC1, RC2) reveals the presence of an additional metastable state, as described in further detail below; similarly, projection onto (RC1, RC3) reveals a range of paths from unfolded to folded. As an aside, we note that the ANTON MD trajectories—as expected—correlate strongly with the predictions of this GDS-derived Koopman model. This is confirmed in Figure 6B, C, and F, which show the MD trajectory data projected onto the GDS/Koopman RCs.

In Figure 7, we have a closer look an interpreting the physical meaning of the RCs identified. The average contact map of regions identified with red boxes are displayed alongside the respective stable regions in both panels of Figure 7. We can see from Panel A, which is the FES expanded in RC1-RC2 space, that RC1 is indeed the unfolded to folded transition. The additional state projected by RC2, appears to be a kinetic misfolded trap. Orthogonal lines to the diagonal on a contact map indicate the location of the β turn residue. The kinetic trap here shows that these β turns are shifted by a residue. Panel A also reveals that this misfolded conformation cannot then enter the folded state, it would need to unravel and unfold to refold. Panel B is a similar FES plot but this time expanded in RC1-RC3 space. Its clear form examining this, there are 2 routes, each of which is corresponds to which β strands connect first. The more dominant route appears to be the folding of the first pair of β strands. The alternative route, appears to contain an extended β strand pair that forms that can hinder the folding process.

**Figure 7:**
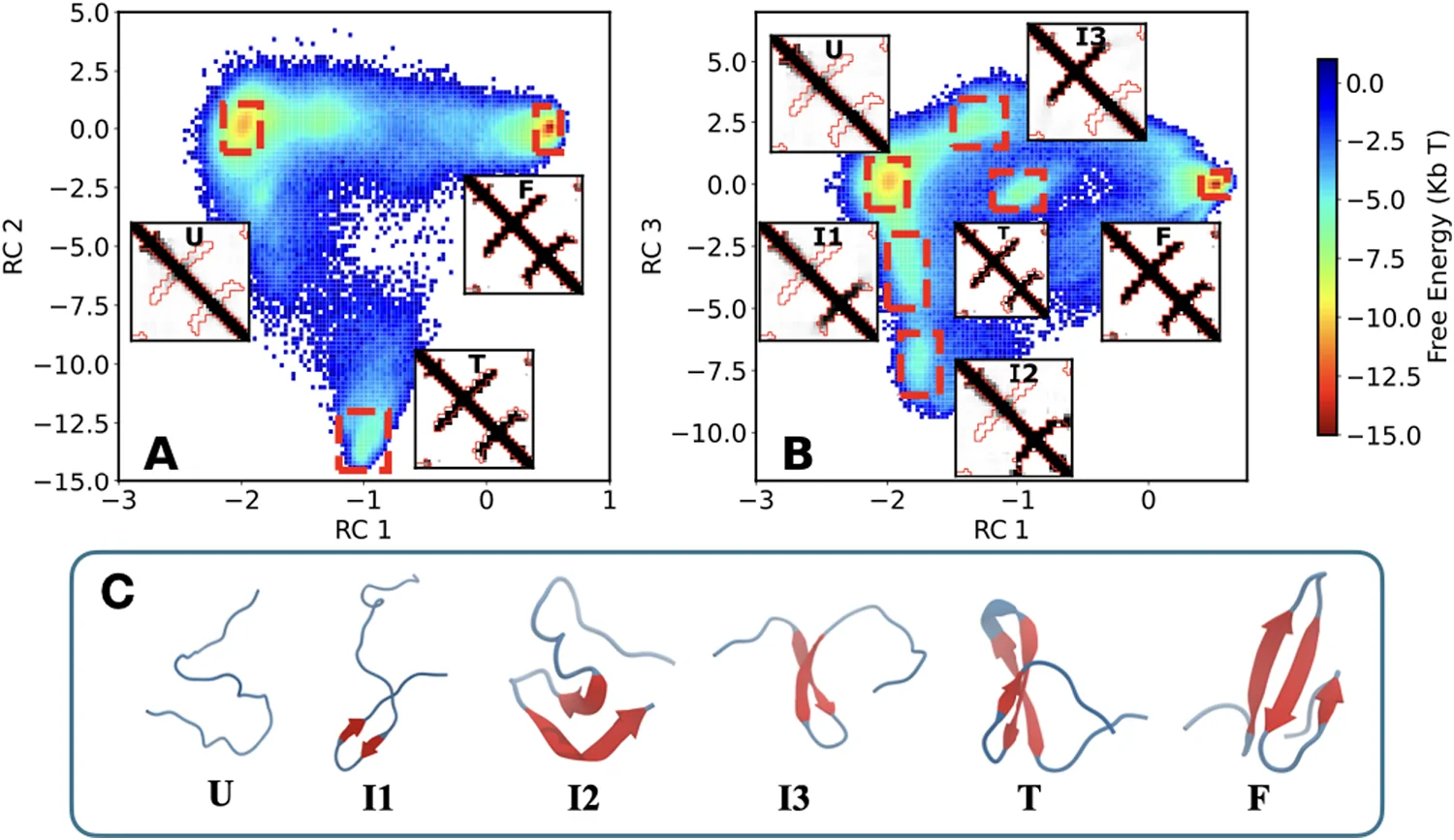
A closer look at the free energy surfaces from panels D and G of Figure 6. Red boxes outline stable regions; corresponding averaged contact maps of the structures inside the boxes are shown nearby, labeled “U”-Unfolded, “T”-Trap, “I”-Intermediate, and “F”-Folded. (A) shows the free energy surface in the (RC1, RC2) plane; we see that RC1 corresponds to the unfolding–folding transition, whereas RC2 distinguishes between a stable trapped state and the rest of the configurational space. Panel (B) shows the free energy surface in the (RC1, RC3) plane, more clearly distinguishing the presence of multiple intermediate states. Finally, panel (C) shows representative structures for each identified state; although cartoon representations are shown highlighting secondary structure, these structures were obtained by back-mapping to fully atomistic, solvated protein configurations.

The GDS-generated Koopman model is very effective in identifying slow-dynamics, and enables further investigation in terms of metastable states and transition pathways. In fact, it is also quite common to use identified RCs from Koopman models to define a discrete state partition and resulting MSM.^18,68^ Specifically, in previous studies of the GTT WW domain data set,^18,68^ a basis of C_α_ and C_β_ pairwise distances was employed (following a range of other previous studies^16,41,69–72^ ), resulting in predicted folding time scales between 6 and 10 μs.^18,67,68^ Curiously, however, we find that the projected modes obtained from our GDS-based model do not correspond well to these previous studies, despite good correspondence of the slowest time scale. Specifically, we find that the time scales for equilibration with the trapped state are much longer than those associated with the folding intermediates, contrary to previous mode projection studies.^18^ The time scales, reported previously^18^ were a result of an MSM built using a 100 ns lagtime after clustering with Koopman modes expanded in the basis of C_α_ and C_β_ pairwise distances; however, those Koopman modes were themselves built with a 10 ns lagtime.

To understand this discrepancy, and provide a more complete comparison between our approach and previous work, we proceeded to build an MSM by discretizing the space spanned by our GDS-informed Koopman modes. Here, we partitioned our Koopman model data using regular space clustering,^10^ a Voronoi partitioning scheme that ensures that no two Voronoi centers lie within a defined threshold. We chose the kinetic threshold to be 40 ns as measured by the commute map^73^ computed using the five slowest modes found from our Koopman model. This threshold was chosen to balance accuracy with number of discrete states, which—in this case—amounted to 232. An implied time scale (ITS) convergence plot is presented in the Supporting Information, showing that 100 ns is a suitable time scales.

From analysis of our simplified MSM, we obtain a folding time scale of 13.8 μs with a standard error of 0.9 μs; this is longer that the 10.2 μs obtained in previous work,^18,68^ even though our discretized MSM only uses 232 states rather than the 1000-state model employed previously. This observation represents a very strongly supportive result for our GDS-based strategy; the variational principle for MSM construction suggests that an optimal approximation of the transfer operator maximizes the time scales (subject to use of a consistent lagtime), so the observation that our MSM exhibits longer time scales than previous studies supports the GDS approach for MSM construction proposed here.

The eigenvectors obtained from our MSM may not necessarily correspond to the Koopman modes identified earlier. To investigate whether the two “slow” processes obtained in our Koopman model correspond to folding-unfolding and trapping, respectively, we visualized the corresponding MSM right-eigenvectors; these act as natural corresponding reaction-coordinates in MSMs. As shown in Figure 8, our interpretation of the slowest modes from our Koopman models aligns well with our MSM. The eigenvector associated with the slowest time scale (Figure 8A) corresponds to the transition between unfolded and folded protein states, whereas the eigenvector associated with the second slowest time scale corresponds to transitions between the trapped and nontrapped states (Figure 8B). Furthermore, we see that our MSM identifies the trapped state as the “kinetically furthest” unfolded state away from the folded state; in other words, our MSM reflects the observation that the trapped state is required to transition back to an unfolded state before proceeding to the folded state, as one would naturally expect.

**Figure 8:**
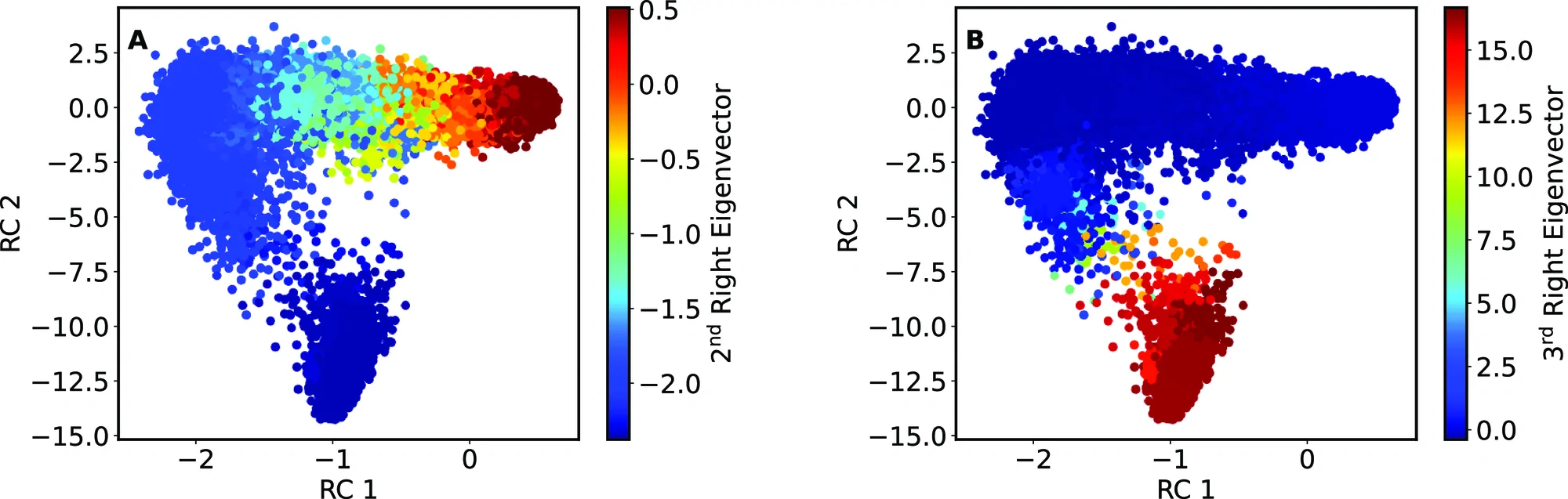
Scatter plots of MD data set projected along the slowest two Koopman modes, RC1 and RC2. Here, points are colored according to to their components in the second and third eigenvector of the MSM, respectively, providing information about the dynamic “flow” between MSM-defined states. The folded state is at (RC1, RC2) = (0.5, 0), the trapped state is at (RC1, RC2) = (−1, −12.5), and the unfolded states lie around (RC1, RC2) = (−2, 0).

To complete the picture of WW domain folding dynamics, we present results of employing the Perron-cluster cluster analysis (PCCA) clustering algorithm^31,74^ on our MSM in Figure 9. The goal of PCCA clustering is to automatically identify a few ‘important’ metastable states from MSMs constructed with many states (such as our MSM built using ∼200 states). This is achieved with a further coarse-graining of an MSM by forming “membership functions” as linear combinations of the MSM indicator basis functions.^31,74^ Given the utility of analyzing contact maps,^49,75^ this provides an interpretable and physically meaningful decomposition of the conformational rates with the relevant kinetics too. As expected, application of PCCA clustering further simplifies the MSM obtained from our original GDS-derived Koopman model, resulting in a reduced model comprising three PCCA states corresponding to the folded, unfolded and trapped regions of configuration space.

**Figure 9:**
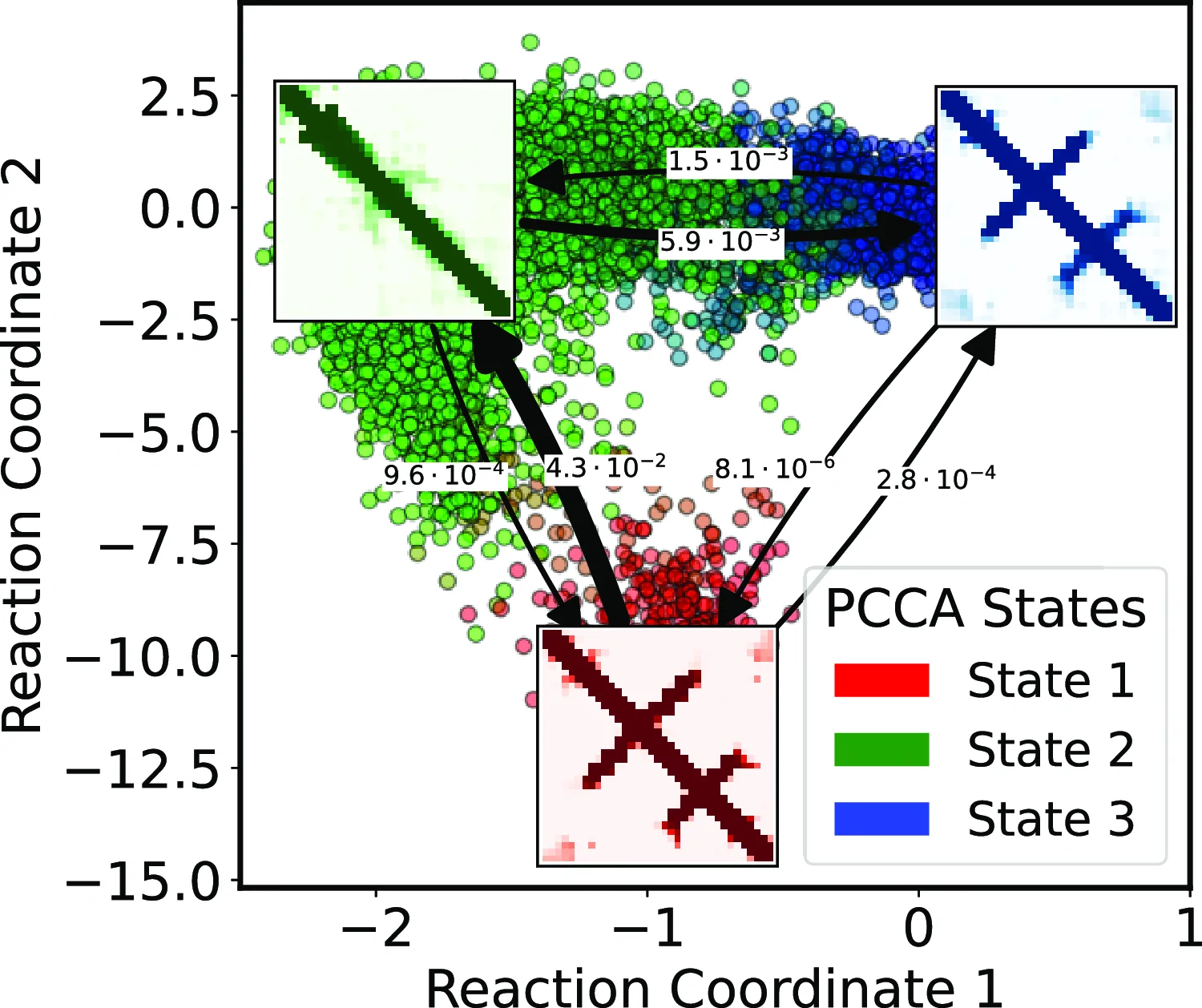
Results obtained after clustering MSM with PCCA. The three states obtained are the trapped, unfolded, and folded states, respectively colored red, green, and blue. Average contact maps for PCCA memberships of over 80% are highlighted with the same colors. Arrows and corresponding labels represent the probability of transition between PCCA states after 100 ns.

### Markov Model of NTL9

To demonstrate the wider applicability of our GDS-based approach, we have repeated the entire simulation and analysis workflow for a separate protein also available in the ANTON data set, namely NTL9. Data for this additional example are shown in the SI; we find that the predicted folding landscapes and observed intermediates align well with previous reduced models. Here, however, we highlight the fact that the estimated folding time-scale of 18.5 μs is in good agreement with previous estimates^15,76^ —with some evidence to suggest that our implied time-scales may represent a variational improvement (as described in the SI, direct numerical estimates for NTL9 folding time-scales are infrequently reported, to the best of our knowledge).

We also provide further comparisons for both proteins between our results and the use of *Q* fraction of native contacts in the SI. We find that *Q* expectedly corresponds well to the slowest Koopman modes for both proteins, but cannot capture the intermediates, traps, and heterogeneity in the pathways. We also report, when using *Q* to define unfolded and folded regions of space, we compute a mean first passage time (MFPT) of of 18 μs for WW Domain and 22 μs for NTL9. These are inline with results from Lindorff-Larsen et al.^6^ which report an MFPT of 21(6) μs for WW Domain and 29(7) μs for NTL9 protein.

## Conclusion

In this work, we have introduced a new strategy for constructing effective basis sets for Markov models by sampling discrete protein-folding pathways, rather than relying on extensive MD simulations. For a fully atomistic (and solvated) protein-folding problem, we have demonstrated how a graph-based representation of the folding landscape—as provided by the simple and efficient GDS algorithm—can be used to identify key intermediate structures in contact-map space. This approach bypasses the need for extensive, pre-existing MD data in basis construction for Markov models, opening a route toward targeted simulations or rate approximations to be performed more efficiently once an informative basis has been established by GDS. We strongly

We first illustrated this strategy using a 2D model that is reminiscent of a rugged protein-folding funnel. We then demonstrated the approach for the folding the GTT variant of the FIP35 WW domain protein in explicit water. The basis sets constructed using GDS were found to provide an accurate and efficient representation of folding kinetics, boosted by the use of the topologically informed SCH distance metric. The resulting Koopman models and MSMs readily identify the important slow dynamical processes, identify key folding intermediates, and even reveal kinetic traps that were not clearly resolved through ‘standard’ order parameters such as the fraction of native contacts. These findings validate the effectiveness of GDS as a complex-landscape exploration strategy, and provide strong evidence for the use of GDS in defining a structurally meaningful basis for Markov models of protein folding. We emphasize that the approach taken here is quite general, and could equally be applied to study protein–protein interaction networks, drug–protein binding, protein misfolding, and other complex transient biophysical processes.

We emphasize again that our focus here is explicitly on addressing step (A)—basis generation (or model construction)—in deploying MSMs, rather than kinetic parametrization (B). As such, kinetic information for GDS-constructed few-state models is obtained using the available ANTON trajectory data, enabling a direct comparison to previous few-state models on a consistent kinetic footing. We restate that, in future studies where a direct comparison is no longer necessary, targetted enhanced sampling methods can utilized effectively with a preconstructed basis.

By using the variational principle for Markov processes, we have shown that our model captures folding dynamics more accurately than previous comparable studies of the GTT variant. In high-dimensional systems, where MD sampling of the entire conformational space is typically not feasible, leveraging our GDS-generated basis becomes advantageous over iterative active sampling methods. Simply put, we can achieve accurate coverage and kinetic connectivity from cheap GDS simulations, rather than extensive MD simulations. A further interesting direction for future research is to consider whether unbiased GDS simulations could be used to automatically generate a set of reaction-coordinates for further biased simulations based on metadynamics, for example, potentially offering a further analysis route for complex free-energy landscapes associated with protein folding and similar processes. The contact map representation is well suited to enhanced sampling for large conformational changes such as exploring intermediates for drug targetting.^77^

The path forward from this work is clear; we now need to develop efficient estimates of free energies and conformational transition rates in the space of contact-maps to effectively extract mechanisms and kinetics for the whole folding process, without relying on exhaustive MD sampling. This is not an intractable problem; free energies of contact maps have been studied previously.^78,79^ Emerging methods for computing committor functions can make calculation of neighboring-contact-map transition rates fast and accessible.^80,81^ Koopman reweighting is now being applied to enhanced sampling trajectories,^24^ which can be utilized effectively with a preconstructed basis. Combining such approaches with GDS is an exciting avenue for future work.

## Supplementary Material



## Data Availability

Code implementing the GDS protein-folding and MSM construction strategy described here can be found at https://github.com/HabershonLab/GDS-fold. This repository includes both Python scripts to run and analyze GDS simulations, as well as scripts to obtain the results plotted in the Figures above. The Anton MD data sets used for model construction were kindly provided by D. E. Shaw Research; further details can be found in ref 6.

## Notes

Further details of Koopman model construction and analysis, as well as further analysis of time scales from Markov models (PDF)
