# Supplementary material for: A Simulation-Free Topological Basis for Building Compact Koopman Models of Protein Folding
Source: J Chem Inf Model. 2026 Sep 1;66(18):12075–89. doi: 10.1021/acs.jcim.6c01905 (PMC13625948; doi:10.1021/acs.jcim.6c01905)
Supplement: Supplementary file 1 [file ci6c01905_si_001.pdf]

Supplementary Information: A simulation-free topological basis for building compact Koopman models of protein folding

Ziad Fakhoury, Gabriele C. Sosso, and Scott Habershon*

Department of Chemistry, University of Warwick, Coventry, CV4 7AL, United Kingdom

E-mail: s.habershon@warwick.ac.uk

Contents

1 Reduced model construction **S3**
1.1 Markov State Model Estimation . . . S3
1.2 The Perron-Frobenius (Transfer) Operator . . . S3
1.3 The Koopman Operator . . . S5
1.4 Estimating Finite Rank Approximation of Koopman Operator . . . S6

2 Toy Model simulations **S7**
2.1 Lagtime Choice . . . S7

3 Protein back-mapping procedure **S7**

4 WW domain protein simulations **S9**
4.1 Koopman Lagtime Choice . . . S9
4.2 Fraction of Native Contacts vs Koopman Modes . . . S10
4.3 SCH vs Hamming Metric . . . S12
4.4 MSM Lagtime Choice . . . S13
4.5 Number of GDS intermediates convergence analysis . . . S13

5 NTL9 protein simulations S14
5.1 Koopman Lagtime Choice S14
5.2 GDS-constructed Markov model for NTL9 domain folding S14
5.3 Fraction of Native Contacts vs Koopman Modes S16
5.4 MSM Lagtime Choice S17

1 Reduced model construction

Here, we present a brief overview of the relevant aspects of constructing Markov state models (MSMs), including Koopman models and estimations.

1.1 Markov State Model Estimation

The P_{ij}^{τ} entries, for a Markov State model can be readily estimated from MD data by evaluating transitions counts between the pre-defined states:

$$C_{ij}^{\tau} := \#(\mathbf{x}(t+\tau) \in A_j \,|\, \mathbf{x}(t) \in A_i).$$

Naively, the transition probability matrix elements can then be estimates as:

$$P_{ij}^{\tau} \approx \frac{C_{ij}^{\tau}}{\sum_k C_{ik}^{\tau}}.$$

However more accurate estimators have been developed where reversibility can be enforced, prior information from experimental data can be used, and uncertainty quantification can be incorporated in building P_{ij}^{τ}.[1–3] Furthermore, MSMs can be built with much shorter lag-times than the equilibration times needed for a typical MD simulation; thus, one can generate a collection of parallel short MD trajectories to efficiently parameterize an MSM.[4]

1.2 The Perron-Frobenius (Transfer) Operator

As mentioned in the Main article text, the problem of building an accurate model of **P** can be reformulated as seeking a finite-rank approximation to the Perron-Frobenius operator:

$$\mathscr{T}[u](x) = \frac{1}{\pi(x)} \int u(y) \Pr(y \to x, \tau) \pi(y) dy, \tag{1}$$

where π is the stationary distribution and $u(x)$ is some distribution of state-space weighted

against the stationary distribution.[5]. This operator is shown to be compact[6] and self-adjoint on $L^2_\pi(\Omega)$ when the dynamical system is reversible; note that $L^2_\pi(\Omega)$ is the Hilbert space of square-integrable functions with inner product $\langle u, v\rangle_\pi := \int_\Omega u(x)v(x)\pi(x)dx$[7]. In general, due to compactness, we can express $\mathscr{T}$

$$\mathscr{T}[u] = \sum_{i=1}^{\infty} \lambda_i \langle \phi_i, u\rangle_\pi \phi_i \tag{2}$$

for eigenfunctions ϕ_i with eigenvalues λ_i. It can be shown that this operator necessarily has an operator norm less than or equal to 1 and thus $|\lambda_i| \leq 1$[7,8]. The eigenvalue of 1 corresponds to the stationary distribution. Just as in the case of MSMs, these eigenvalues are related to relaxation times via $t_i = -\tau/\ln\lambda_i$, and the associated eigenvectors describe the collective modes through which the system relaxes toward equilibrium. Furthermore, mild assumptions[7] that are assumed true for molecular dynamics systems, suggest we can break down the modes of $\mathscr{T}$ into fast and slow modes:

$$\mathscr{T}[u] = \sum_{i=1}^{N} \lambda_i \langle \phi_i, u\rangle_\pi \phi_i + \mathscr{T}^{fast} u \tag{3}$$

where the spectrum of $\mathscr{T}^{fast}$ is negligibly small seperated by a significant spectral gap, and can be truncated in the interest of long timescale dynamics.

If one restricts themselves to a finite-rank approximation with a basis of indicator functions over non-overlapping sets $\{A_i\}$, then we have that

$$\langle \mathbb{1}_{A_i}, \mathscr{T}\mathbb{1}_{A_j}\rangle_\pi = \sum_k T_{jk} \langle \mathbb{1}_{A_i}, \mathbb{1}_{A_k}\rangle_\pi = T_{ji}\pi(A_i)$$

$$T_{ji} = \frac{\langle \mathbb{1}_{A_i}, \mathscr{T}\mathbb{1}_{A_j}\rangle_\pi}{\pi(A_i)} = \frac{1}{\pi(A_i)} \int \mathbb{1}_{A_i}(x) \left(\frac{1}{\pi(x)} \int \mathbb{1}_{A_j}(y) \Pr(y \to x)\pi(y)dy \right) \pi(x)dx$$

$$= \frac{1}{\pi(A_i)} \int_{x\in A_i} \int_{y \in A_j} \Pr(y \to x)\pi(y)dydx = \frac{1}{\pi(A_i)} \int_{x\in A_i} \int_{y \in A_j} \Pr(x \to y)\pi(x)dydx$$

$$= \Pr\left(x(\tau) \in A_j \,|\, x(0) \in A_i\right) = P_{ij}$$

Therefore, one can see that this Perron-Frobenius operator is simply a generalisation of a Markov State Model for general basis sets as opposed to indicator functions.

1.3 The Koopman Operator

The Koopman operator for time τ evolution is defined as

$$\mathscr{K}[f](x) = \int f(y)\,\Pr(x \to y, \tau)dy$$

This is interpreted as the expectation of the value of an observable f when the system progressed from a state x after time τ. The Koopman operator does not require a reference to the equilibrium distribution, which makes it advantageous to the Transfer operator when considering data driven methods and non-equilibrium systems. Wu et al.[9] have shown that maximising the singular values of the Koopman operator gives a similar variational and decomposition expression to Eq. 2. In general, MD simulation data can be far from equilibrium, especially when aggregating short MD trajectories. In this case, we can treat MD simulation data a set of transition pairs $\{(x_t, x_{t+\tau})\}$, where $x(t)$ are distributed across some empirical distribution ρ_0 and $x_{t+\tau}$ is sampled from ρ_1 where

$$\rho_1(x) = \int \rho_0(y)\,\Pr(y \to x, \tau)dy$$

In this case, the Koopman operator can be considered a map from functions defined on $L^2_{\rho_1}(\Omega)$ to functions $L^2_{\rho_0}(\Omega)$. The adjoint of this, $\mathscr{K}^*$, can be elucidated when considering the following calculation.

$$\langle f, \mathscr{K} g\rangle_{\rho_0} = \int \rho_0(x) f(x) \mathscr{K} g(x) dx = \int \rho_0(x) f(x) \left(\int g(y)\,\Pr(x \to y, \tau)dy \right) dx$$

$$= \int\int \rho_0(x)\,\Pr(x \to y, \tau) f(x) dx\, g(y) dy = \int \left(\frac{1}{\rho_1(y)} \int \rho_0(x)\,\Pr(x \to y, \tau) f(x) dx \right) g(y) \rho_1(y) dy$$

$$= \langle K^* f, g\rangle_{\rho_1}$$

This suggest $\mathscr{K}^* \cdot = \mathscr{T} \cdot = \frac{1}{\rho_1(y)} \int \rho_0(x) \Pr(x \to y, \tau) \cdot dx$. Notice that if we were sampling from the stationary distribution, $\pi(x)$, then the adjoint is indeed the Perron-Frobenius Operator. If the dynamics are reversible (detailed balance), we know that $\pi(x) \Pr(x \to y) = \pi(y) \Pr(y \to x)$ we can show that $\mathscr{T} = K^* = K$

$$\mathscr{T}[f](y) = \int f(x) \Pr(x \to y) \frac{\pi(x)}{\pi(y)} dx = \int f(x) \Pr(y \to x) \frac{\cancel{\pi(y)}}{\cancel{\pi(y)}} dx = \mathscr{K}[f](y)$$

1.4 Estimating Finite Rank Approximation of Koopman Operator

Given a selected basis $\{\chi_i\}$, a finite rank approximation of a Koopman operator is statistically estimated from MD data transition pairs $(x_t, x_{t+\tau})$. To see how to arrive at this statistical estimate, a Galerkin projection of $\mathscr{K}$ is used with respect to the equilibirum weighted inner product. In other words, we seek $K = (K_{ij})$ such that

$$\langle \chi_i, (K - \mathscr{K}) \chi_j \rangle_\pi = 0$$

By proceeding with this we find

$$\langle \chi_i, \mathscr{K} \chi_j \rangle_\pi = \sum_k \langle \chi_i, K_{kj} \chi_j \rangle_\pi \implies C^{0t} = C^{00} K$$

Where the covariance matrices are $C^{0t} = \langle \chi_i, \mathscr{K} \chi_j \rangle_\pi$ and $C^{00} = \langle \chi_i, \chi_j \rangle_\pi$. Writing the explicit integral we can quickly see how to arrive at a Monte Carlo estimate

$$C^{00}_{ij} = \int \chi_i(x) \chi_j(x) \pi(x) dx \approx \frac{1}{N} \sum_n \chi_i(x(t_n)) \chi_j(x(t_n)) \tag{4}$$

$$C^{0t}_{ij} = \int \int \chi_i(x) \Pr(x \to y) \chi_j(y) \pi(x) dx dy \approx \frac{1}{N} \sum_n \chi_i(x(t_n)) \chi_j(x(t_n + \tau)) \tag{5}$$

2 Toy Model simulations

2.1 Lagtime Choice

Before building a Markov model, we selected the lagtime τ. Typically, τ is chosen to capture slow processes and equilibrate fast processes; in other words, the lagtime τ should be selected so that the system behaves in a Markovian fashion. However, no reliable method currently exists to achieve this *a priori*. Instead, we opt here to us a simple heuristic method derived from observing the extent to which a configuration drifts (on average) after different lagtimes, as shown in Fig. S1. Here, the displacement over a chosen timescale τ is calculated as $|r(t+\tau) - r(t)|$ using frames in our Langevin MD simulations. For lagtimes that are too fast to enable sufficient equilibration, we observe a narrow distribution of small displacements, indicating that configurations are highly-localized over the (short) timescale τ. As τ increases, the density distribution for displacements broadens significantly, indicating more widespread equilibration across a range of configurational states. We find that, for values $\tau \geq 1.5$, the displacement density distribution is broadly similar; as such, we select a lagtime of $\tau = 1.5$ time-units in the following analysis.

When a typical implied timescale analysis using the GDS basis Markov model, presented in Fig. S2, we find this lagtime is indeed suitable and results in approximately Markovian dynamics.

3 Protein back-mapping procedure

In all calculations reported here, for both the WW domain protein and NTL9, fully-atomistic representation of the full system were obtained using the CHARMM36m-TIP3 forcefield[10]. The three-dimensional protein-solvent structure was reconstructed using a "back-mapping" procedure. As described in the main text, we first obtain a biased structure in the fully-atomistic protein-water system that is consistent with the current target contact map. This is achieved by running a high temperature MD simulation with a strong graph-restraining potential (GRP) bias. We subsequently cool this configuration (still applying the GRP bias) to obtain a more thermodynamically-stable

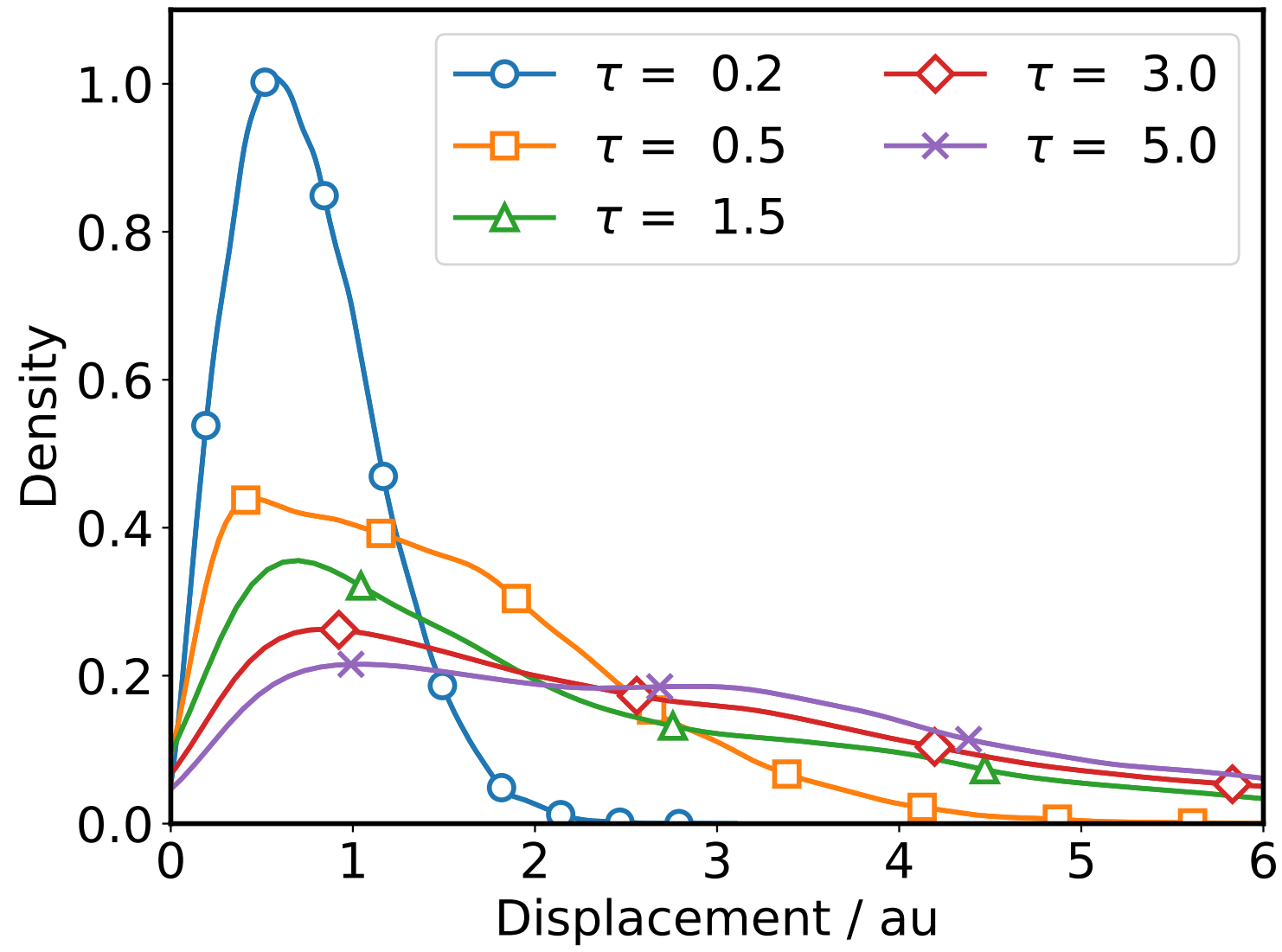

Figure S1 Kernel density estimation plot of displacement after Langevin MD propagation for different lagtimes $\tau = [0.2, 0.5, 1.5, 3.0, 5.0]$. The system exhibits a narrow displacement distribution for $\tau < 1.5$ time-units, slowing substantially for longer lagtimes τ - as demonstrated by the similar displacement distributions.

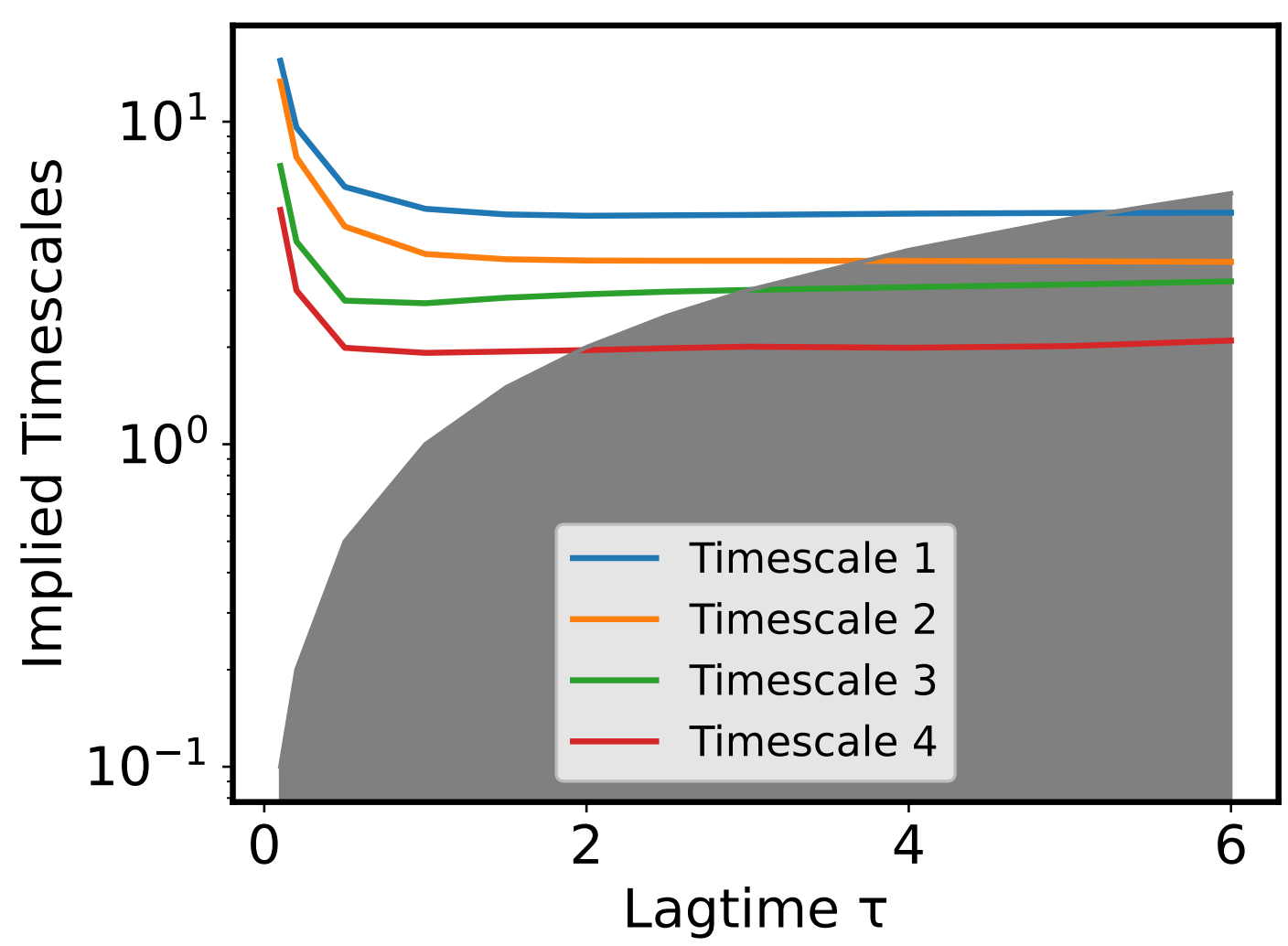

Figure S2 Implied timescale plot for the toy for the Markov Model with the GDS derived basis. We see that indeed, the dynamics become approximately Markovian at lagtimes 1.5 time-units or higher.

representative structure.

The folded protein was solvated using the CHARMM-GUI[11] solution builder feature, with KCl ion concentration of 0.15M. The simulation box used for these simulations was cubic with

box-length 62 Å, allowing for large conformational changes without significant finite-size effects. With the protein geometry positionally-restrained (using a harmonic potential with force constant $40 kJ\text{mol}^{-1}\text{nm}^{-2}$), the solvent geometry was initially minimised, with a force tolerance of $1000 kJ\text{mol}^{-1}\text{nm}^{-1}$. Subsequently, using the standard leap-frog MD integrator from GROMACS, we equilibrated the protein in the canonical ensemble for 125 ps at 303.15 K using the Bussi-Donadio-Parrinello thermostat[12] with a coupling constant of 1ps. The solvated protein structures obtained were subsequently used as the starting point to obtain representative ”back-mapped” protein-solvent structures. To quickly decorrelate from the starting structure and overcome any PES barriers induced by the interatomic interactions, we simulated this system with the GRP in isothermal-isobaric conditions at a temperature of $T = 603.15$K and pressure $p = 1.0$ bar for 100 ps. Here we used both stochastic velocity rescaling and isotropic cell rescaling[13] with time-constants of 1 ps and 5 ps, respectively. We then set the thermostat to a reference temperature of 303.15 K to relax the structure (with GRP restraints still enforced) for a further 100 ps. A final 1 ns run was then performed without GRP restraints; this ensures that the contact-maps formed were thermodynamically stable (or, at worst, metastable) and as structurally similar as possible to the original target contact map for structure generation. The outcome of this procedure is generation of representative protein-solvent structures that corresponding to each contact-map obtained by GDS mapping of the protein-folding landscape.

4 WW domain protein simulations

4.1 Koopman Lagtime Choice

As noted above, fitting the Koopman operator requires selection of an appropriate lagtime over which the ‘fast’ degrees-of-freedom in the system effectively equilibrate. We perform a similar analysis to that presented for the 2D model potential above, but using the ANTON MD trajectories to provide transient input data. The results are illustrated in Fig. S3, from which it is clear that a lagtime of $\tau = 100$ ns represents an appropriate choice. Specifically, we find that shorter lagtimes

exhibit a distance-density distribution that is still time-dependent; however, beyond $\tau = 100$ ns, only negligible changes in the distance-density distribution are observed.

When a typical implied timescale analysis using the GDS basis Markov model, presented in Fig. S4, we find this lagtime is indeed suitable and results in Markovian dynamics.

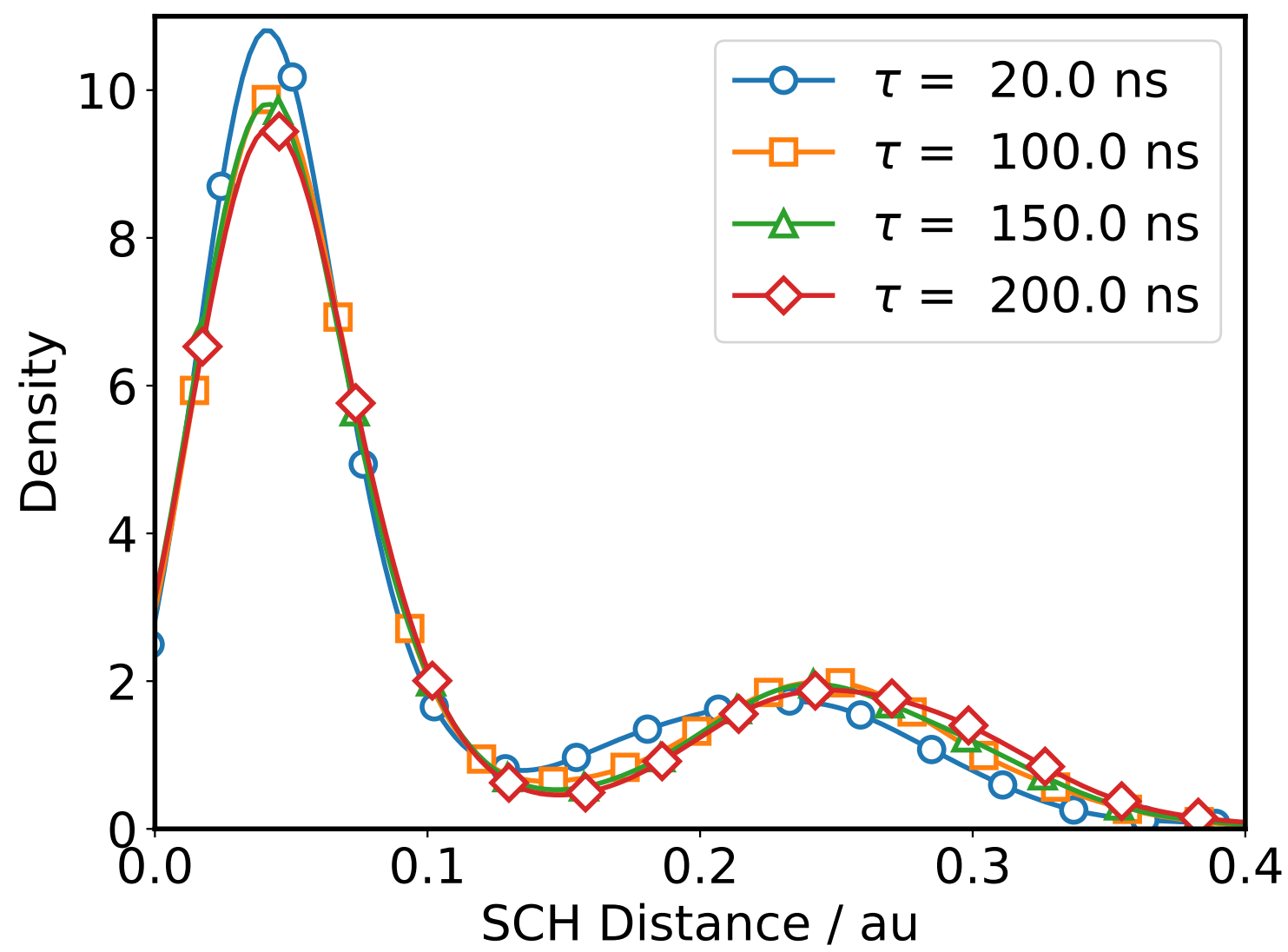

Figure S3 A KDE plot of the SCH distances between uniformly random sampled structures from the ANTON MD trajectories and structures after a lagtime $\tau = [20, 100, 150, 200]ns$. For lagtimes greater than 100 ns, there is little change in the displacement-density distribution, suggesting sufficient equilibration over fast processes.

4.2 Fraction of Native Contacts vs Koopman Modes

The fraction of native contacts is a commonly used reaction coordiante to track the progress of protein folding. Simply using the fraction of native contacts, Q, as a reaction coordinate for folding has been shown to have severe non-Markovian artifacts[14–16]. It also lacks structure specificity; a particular Q can be obtained from several topologically-distinct conformations, such that this approach may struggle to resolve multiple pathways or trapped intermediates. In Fig. S5, we compare the fitted GDS-derived Koopman modes for the WW domain against Q. We find that Q expectedly corresponds well to the slowest Koopman modes for both proteins, but cannot capture the intermediates, traps, and heterogeneity in the pathways.

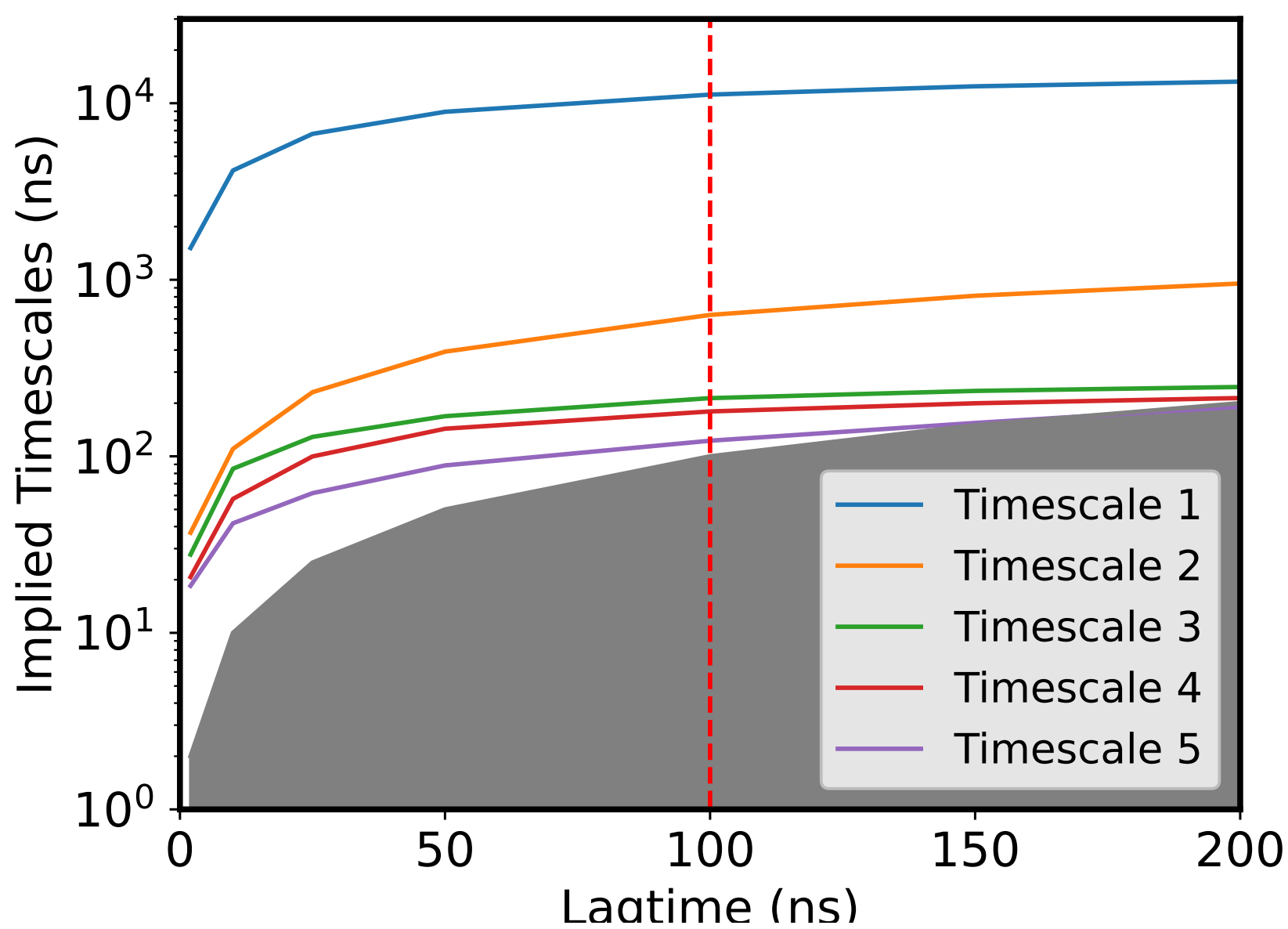

Figure S4 Implied timescale plot for the koopman projection with the GDS derived basis. We see that indeed, the dynamics become approximately Markovian at lagtimes 100ns or higher.

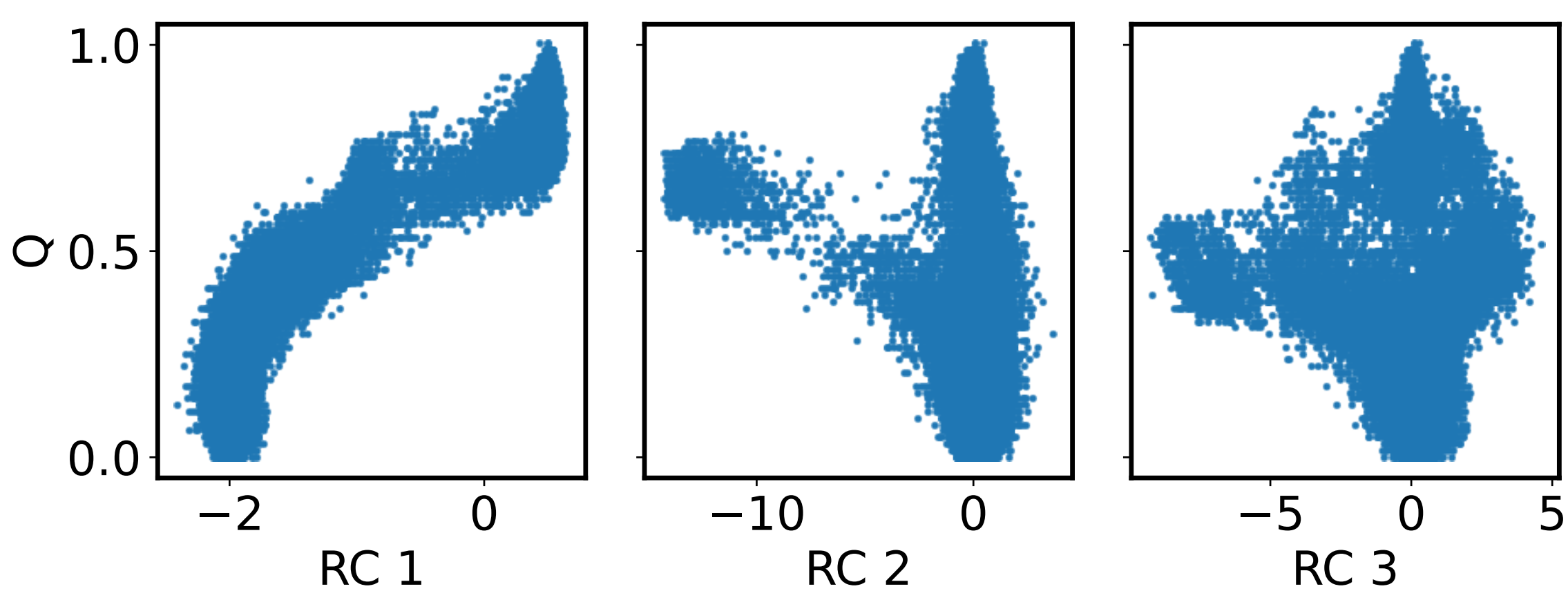

Figure S5 Scatter plots for the fraction of native contacts Q against the Koopman modes found for the WW Domain protein. We find that as expected, RC1 corresponds well with Q, but Q cannot capture the intermediates, traps, and heterogeneity in the pathways.

4.3 SCH vs Hamming Metric

The utility of the SCH metric, introduced and discussed in previous work,[17] is further presented here. As a comparison between using the Hamming metric (the normalised number of incorrect contacts) and the SCH metric, we compare how well the metric can express a significant structural transition after a given lagtime. We see in Fig. S3, there is a cleaner separation of 2 modes in the displacement-density distribution when using the SCH metric than with the Hamming Metric in Fig. S6. This suggests that using the SCH metric is a much more reliable method to determine whether a significant long-time structural transition has taken place.

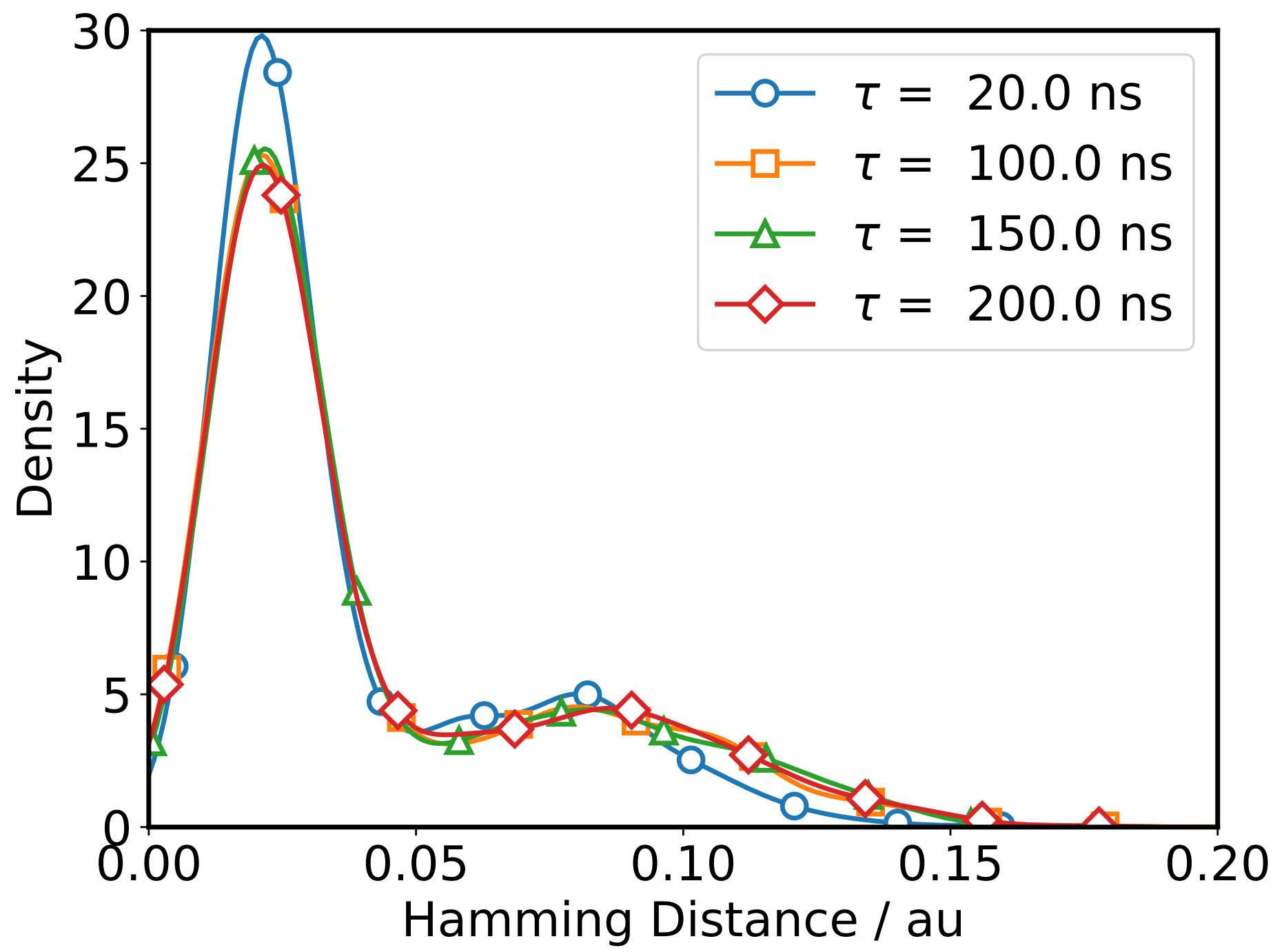

Figure S6 A KDE plot of the Hamming distances between uniformly random sampled structures from the ANTON MD trajectories and structures after a lagtime $\tau = [20, 100, 150, 200] ns$. Unlike the SCH metric, we cannot make a clean distinction between the 2 distributions centred around modes. As we have seen in Fig. S3, for lagtimes greater than 100 ns, there is little change in the displacement-density distribution. This supports sufficient equilibration over fast processes when choosing lagtimes greater than 100 ns.

4.4 MSM Lagtime Choice

An Implied Timescale (ITS) convergence plot was used to assess a suitable lagtime to ensure that the Markovian assumption holds; satisfyingly, as shown in Fig. S7, we find that a lagtime of 100 ns is indeed sufficiently long to obtain converged timescales, in agreement with the analysis shown in Fig. S3.

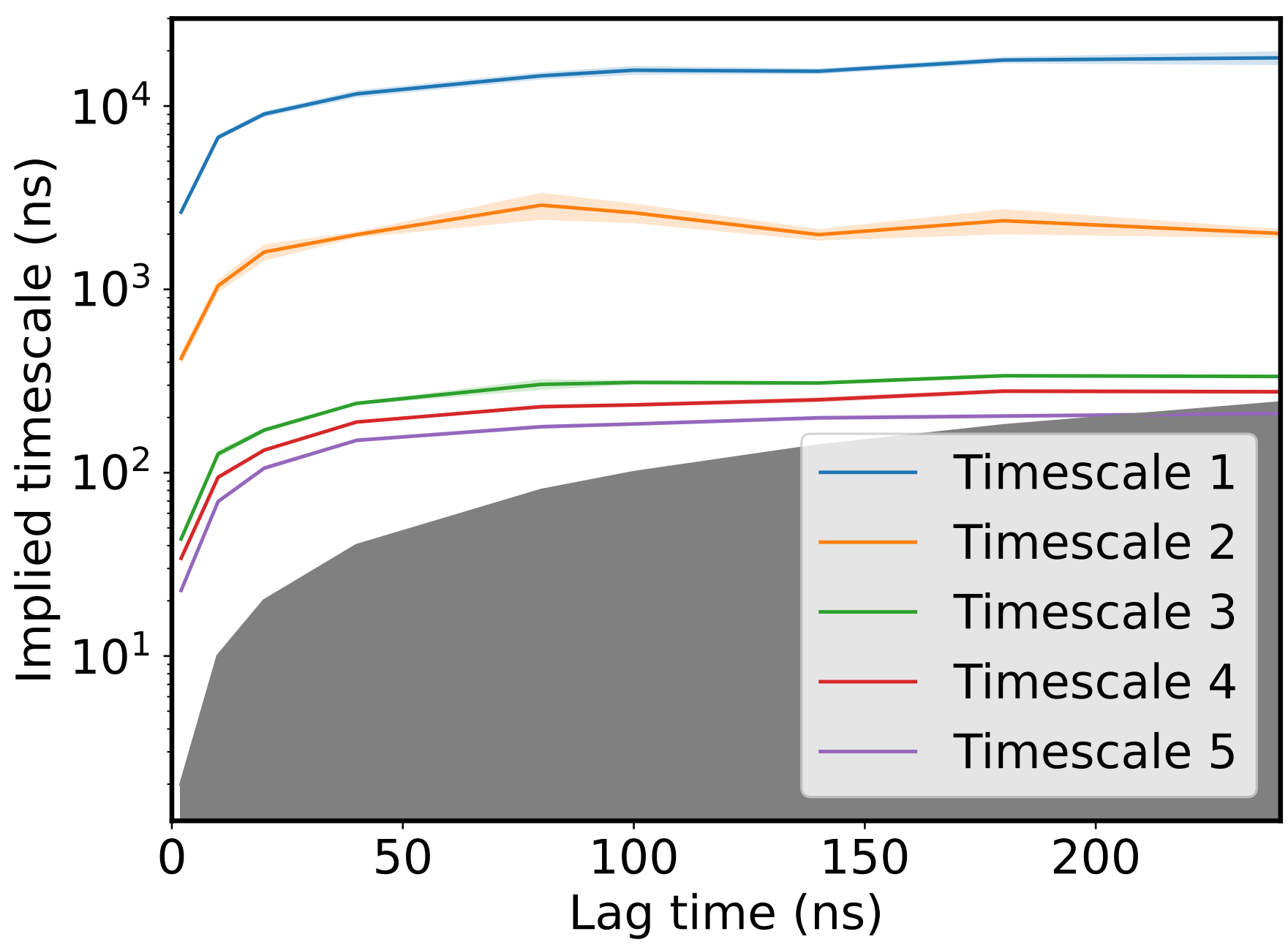

Figure S7 Implied timescale (ITS) convergence plot for an MSM built with regular space clustering on our WW Domain Koopman projected data. We find that a lagtime of 100 ns is sufficient to enable evaluation of converged timescales. We also observe two separate relaxation timescales (blue and orange) that are distinct from the remaining dynamics. Standard errors were measured by bootstrapping; specifically block sub trajectories where sampled randomly with blocks of size 20µs to ensure decorrelation. Error bars which are $\pm$ the standard error are plotted.

4.5 Number of GDS intermediates convergence analysis

In the main article, 100 GDS paths of length $N = 10$ were used to yield 800 physically-relevant intermediate contact maps. This set was further pruned to 608 basis functions by removing closely-similar structures; specifically, a structure was removed if it had an SCH distance of less than

0.1 to any other retained structure. To assess whether this basis provides sufficient coverage, we generated an extended set of 200 GDS paths, yielding 1600 physically-relevant intermediates and, following the same pruning procedure, a total basis size of 834.

Figure S8 shows the convergence of the implied timescales with respect to the number of retained basis functions, using a fixed Koopman lagtime of 100 ns. For each basis size, multiple random subsets were selected from the complete set of 834 basis functions and a separate Koopman model was constructed for each subset. The plotted timescales correspond to the mean over these random sub-basis selections, with the shaded regions indicating $\pm$ one standard deviation. As the basis size is increased, the slow implied timescales become increasingly insensitive to the particular subset selected, demonstrating convergence of the dominant kinetic modes with respect to the size of the GDS-derived basis. We note, that the second time-scale seems to have room for further improvement, but since the Koopman modes are then used to build an MSM which is more expressive and simplified, we point to the results of MSM performance with just 232 states (indicator basis) that are built with Koopman modes using only 608 basis functions.

5 NTL9 protein simulations

5.1 Koopman Lagtime Choice

We perform a similar analysis to that presented for the WW domain protein, but instead do this for NTL9. The results are illustrated in Fig. S9, from which it is clear that a lagtime of $\tau = 150$ ns represents an appropriate choice similar as the above protein.

5.2 GDS-constructed Markov model for NTL9 domain folding

100 GDS runs were initiated starting from a ‘null’ contact-map representing an entirely-unfolded configuration of the protein sequence; the contact-map of the folded protein was used as the target end-point for all GDS trajectories. The GDS graph sequences were chosen to be of length $N = 10$,

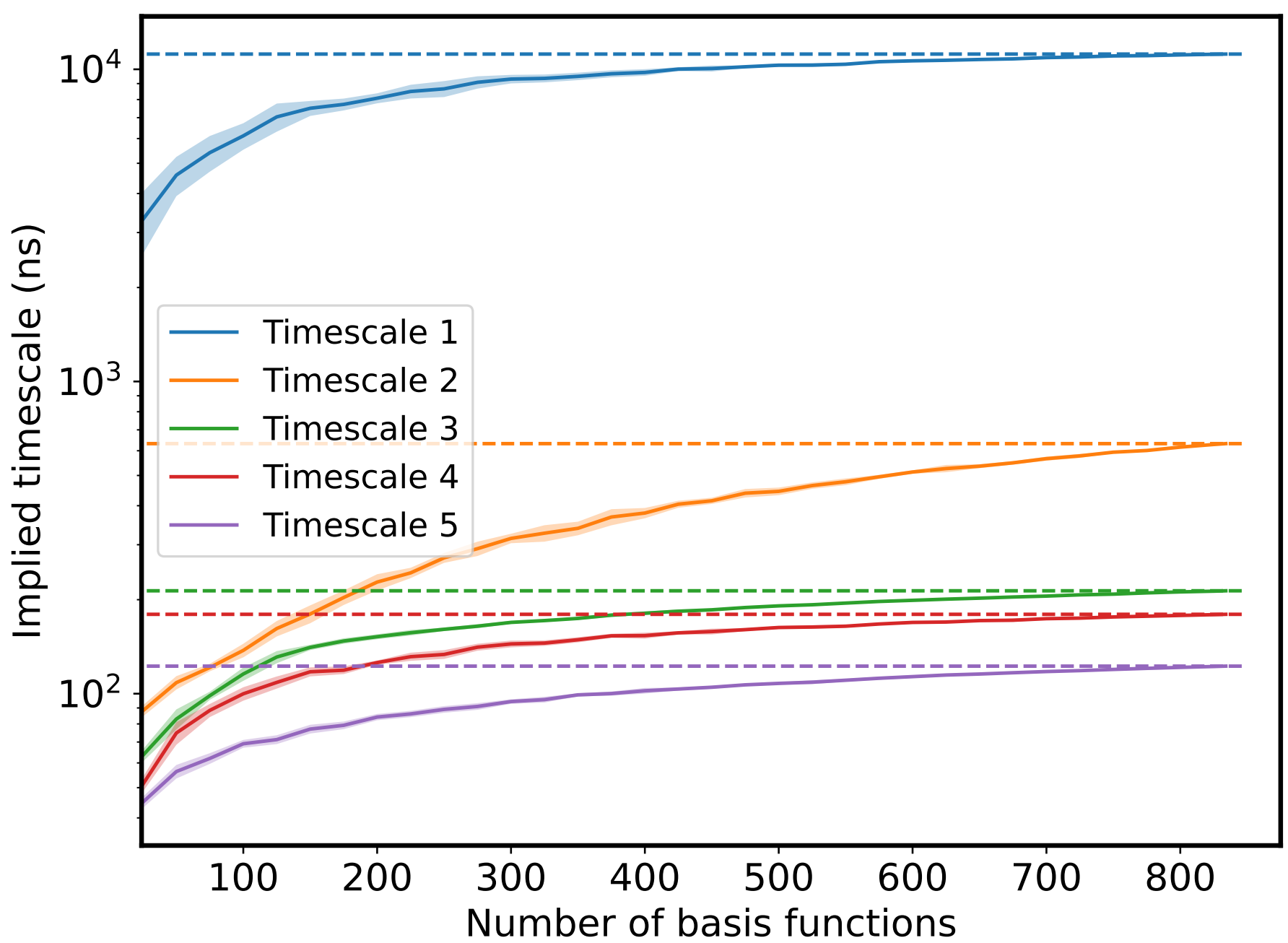

Figure S8 Convergence of the leading implied timescales of the WW domain Koopman model with respect to the number of GDS-derived basis functions, evaluated using a fixed lagtime of $\tau = 100$ ns. For each basis size, random subsets were drawn from the complete set of 834 basis functions and independent Koopman models were constructed. Solid lines show the mean implied timescales over the sampled sub-bases, while shaded regions indicate $\pm$ one standard deviation, quantifying the sensitivity of the estimated timescales to the particular choice of retained basis functions.

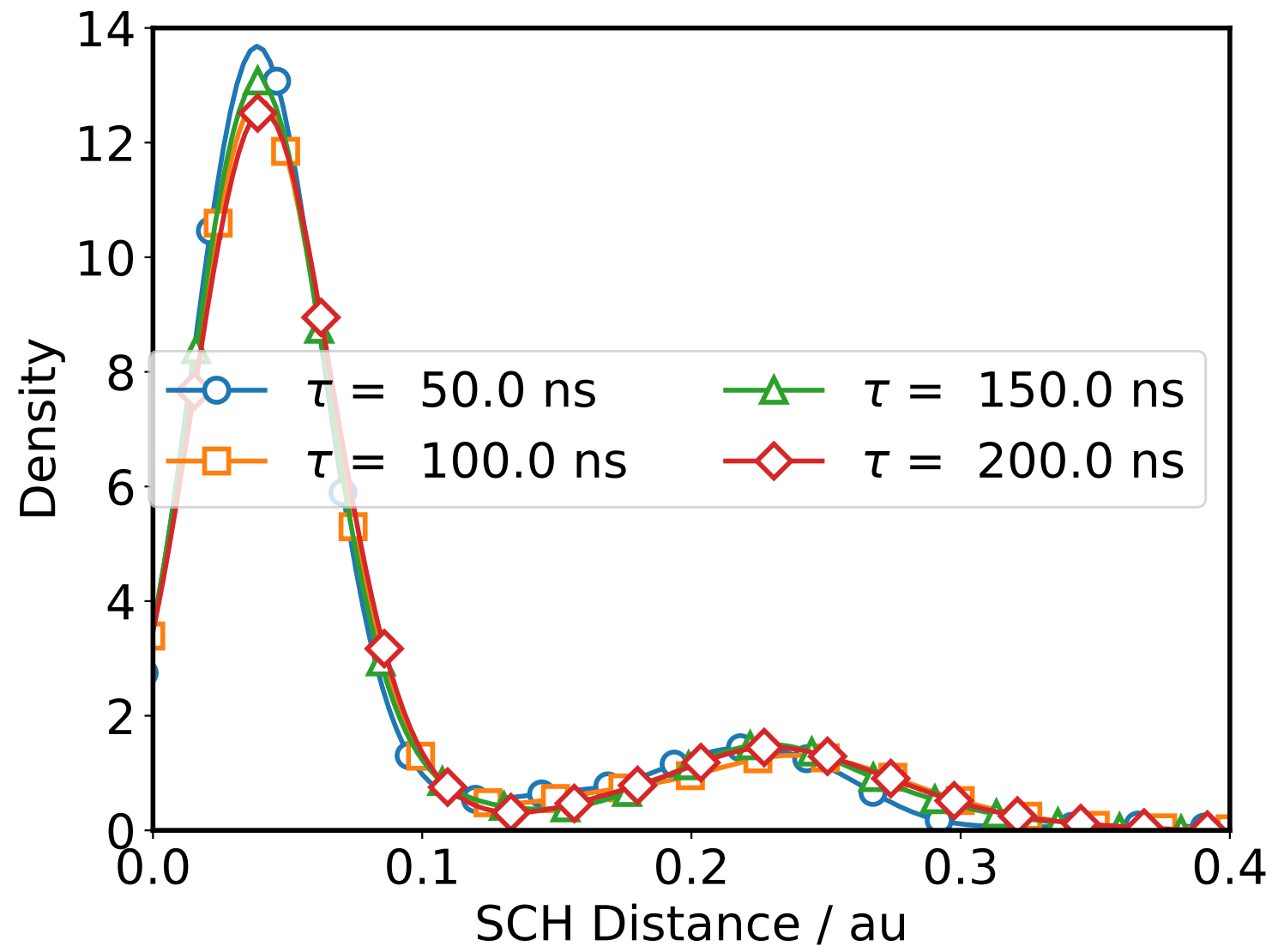

Figure S9 A KDE plot of the SCH distances between uniformly random sampled structures from the ANTON MD trajectories and structures after a lagtime $\tau = [50, 100, 150, 200]ns$. For lagtimes greater than 150 ns, the displacement-density distribution stays constant, suggesting sufficient equilibration over fast processes.

inclusive of unfolded and folded endpoints, with the parameter $k_{cont} = 0.05$ chosen like the WW domain protein. This choice was made based on Figure S9 , where it is clear that structures within this distance are kinetically accessible. Our SA procedure employed is the exact same for th WW domain described in the main text.

A Koopman operator represented in the GDS basis was subsequently fit with that lagtime. Here, we generated a Koopman model describing the three slowest relaxing modes of interest. The resulting timescales were 16.7 µs, 0.7 μs and 0.4 µ s, computed by $t_i = -\frac{\tau}{\ln(\lambda)}$, where τ is the lagtime. The projections of the MD data along these three slowest Koopman modes are shown in Fig. S10.

5.3 Fraction of Native Contacts vs Koopman Modes

The fraction of native contacts is a commonly used reaction coordiante to track the progress of protein folding. Simply using the fraction of native contacts, Q, as a reaction coordinate for folding has been shown to have severe non-Markovian artifacts[14–16]. It also lacks structure specificity;

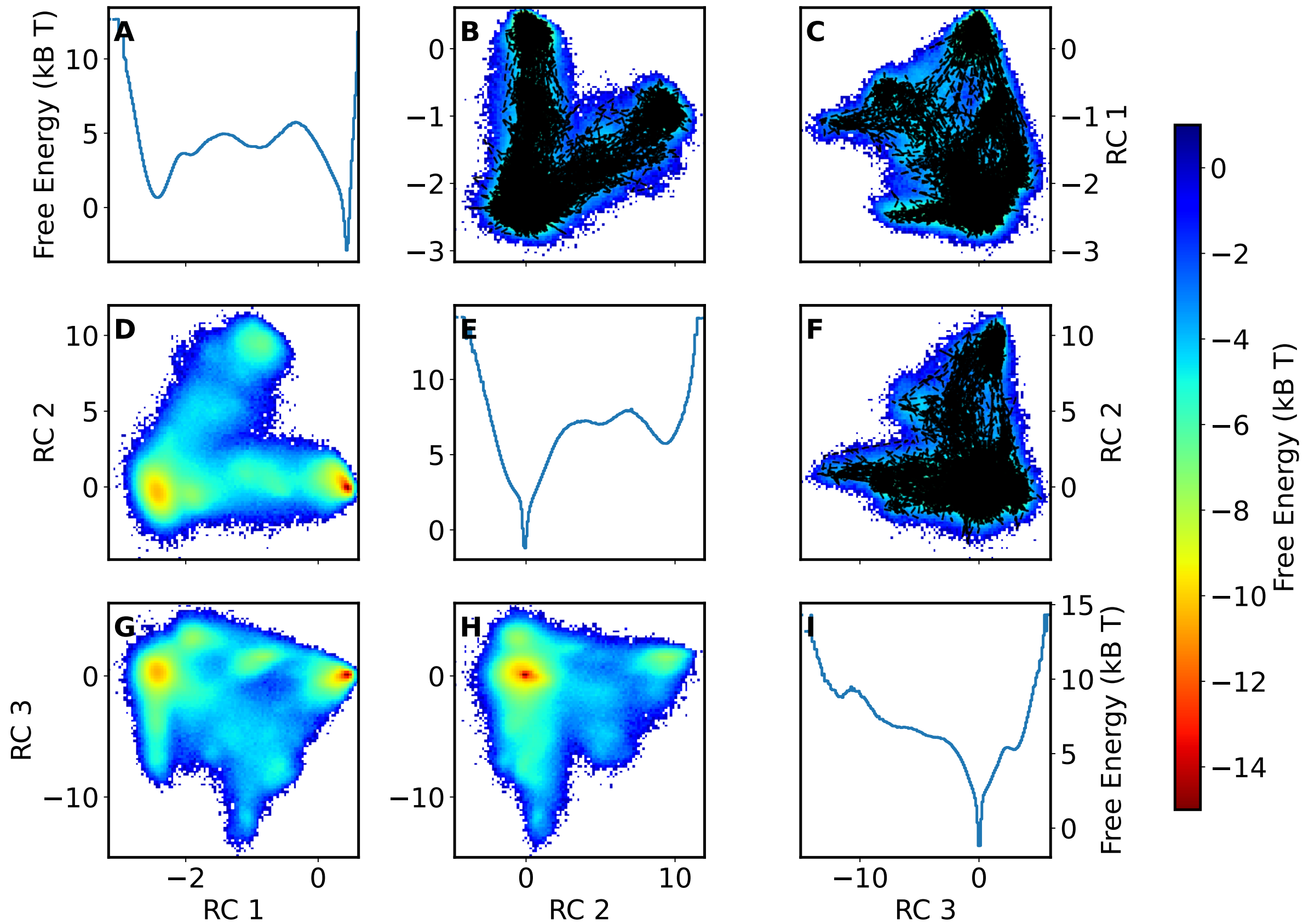

Figure S10 Free energy projections of the NTL9 protein along the three slowest modes, RC1-3. Along the diagonal, panels {A,E,I}, are the free energy curves along the separate reaction coordinates. The off-diagonal panels show 2D Free energy surfaces projected along two of the modes. Panels D, G and H are the free energy surface plots for RC2 against RC1, RC3 against RC1, and RC3 against RC2, respectively. The upper triangular off-diagonal panels B, C and F are reflections of panels D, G and H, respectively, but with samples of the MD trajectory data overlaid.

a particular Q can be obtained from several topologically-distinct conformations, such that this approach may struggle to resolve multiple pathways or trapped intermediates. In Fig. S12, we compare the fitted GDS-derived Koopman modes for the NTL9 protein against Q. We find that Q expectedly corresponds well to the slowest Koopman modes for both proteins, but cannot capture the intermediates, traps, and heterogeneity in the pathways.

5.4 MSM Lagtime Choice

An Implied Timescale (ITS) convergence plot was used to assess a suitable lagtime to ensure that the Markovian assumption holds; satisfyingly, as shown in Fig. S13, we find that a lagtime of 150

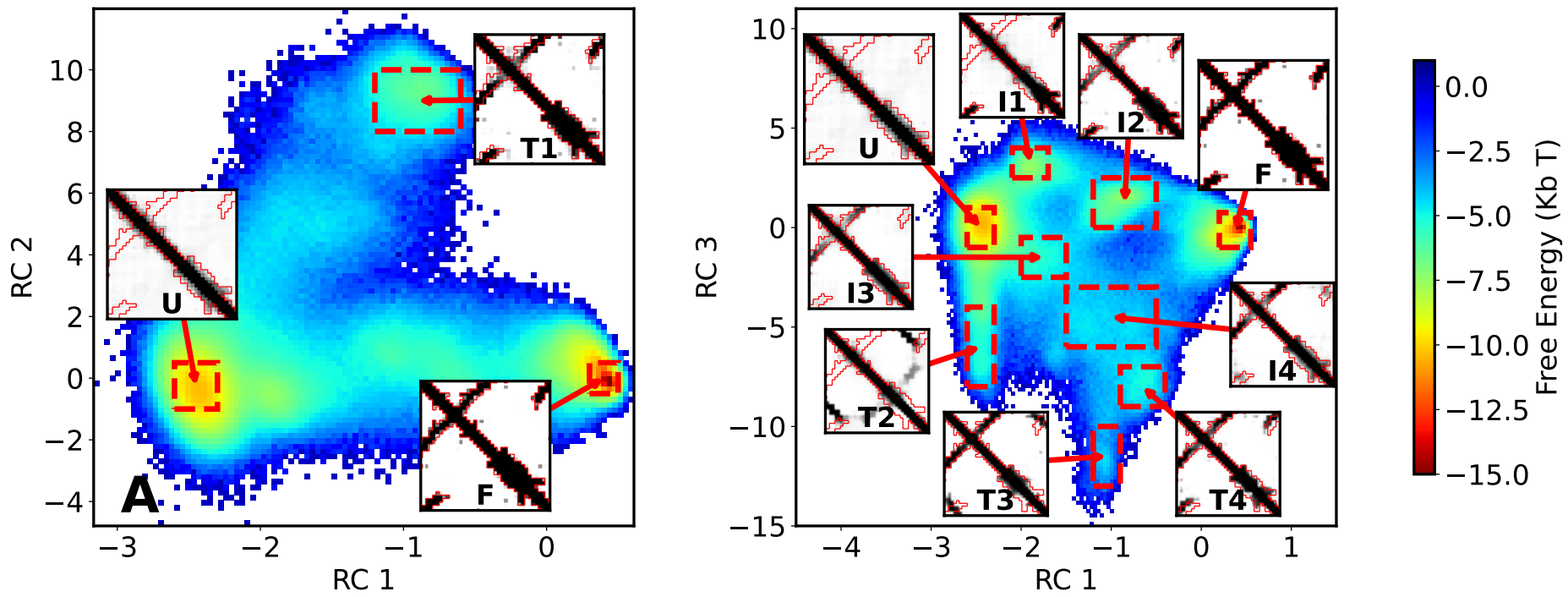

Figure S11 A closer look at the free energy surfaces from panels D and G of Fig. S10. Red boxes outline stable regions; corresponding averaged contact maps of the structures inside the boxes are shown nearby, labelled "U"-Unfolded, "T"-Trap, "I"-Intermediate, and "F"-Folded. (A) shows the free energy surface in the (RC1,RC2) plane; we see that RC1 corresponds to the unfolding-folding transition, whereas RC2 distinguishes between a stable trapped state and the rest of the configurational space. Panel (B) shows the free energy surface in the (RC1,RC3) plane, more clearly distinguishing the presence of multiple intermediate and trapped states.

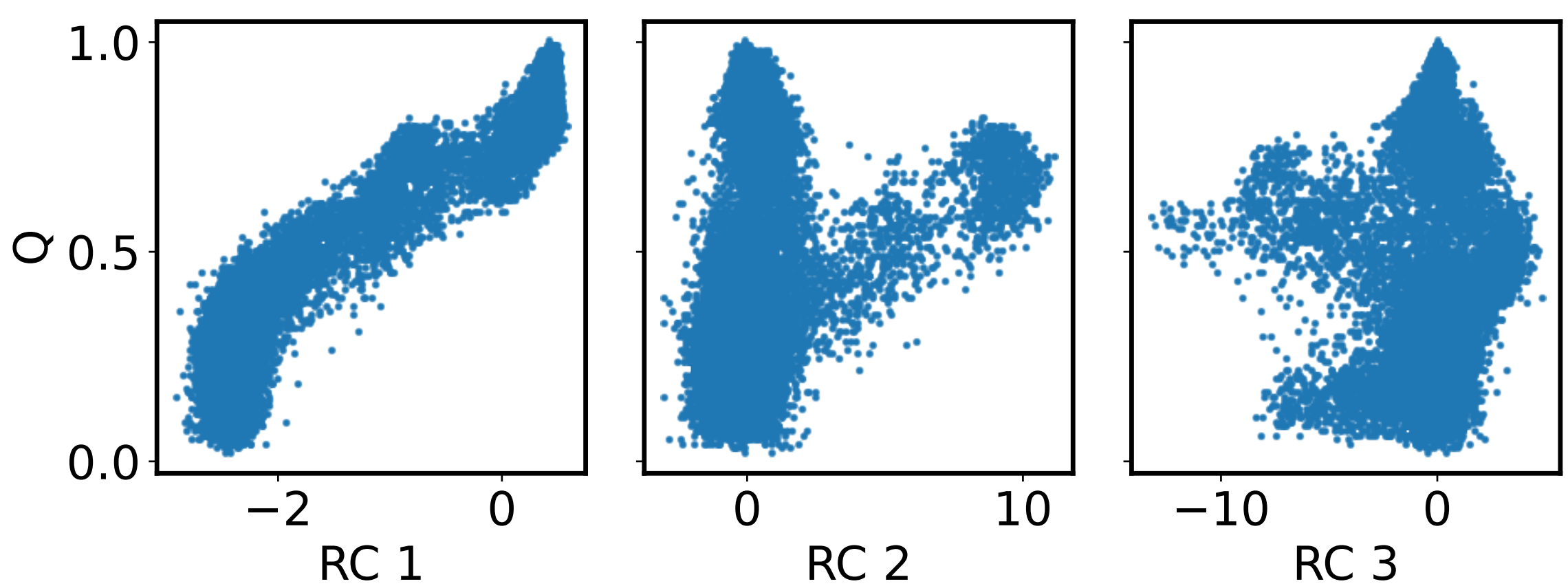

Figure S12 Scatter plots for the fraction of native contacts Q against the Koopman modes found for the NTL9 protein. We find that as expected, RC1 corresponds well with Q, but Q cannot capture the intermediates, traps, and heterogeneity in the pathways.

ns is indeed sufficiently long to obtain converged timescales, in agreement with the analysis shown in Fig. S9.

From analysis of this MSM, with 476 states obtained with regular space clustering, we obtain a folding timescale of 18.5μs with a standard error of 0.7μs.

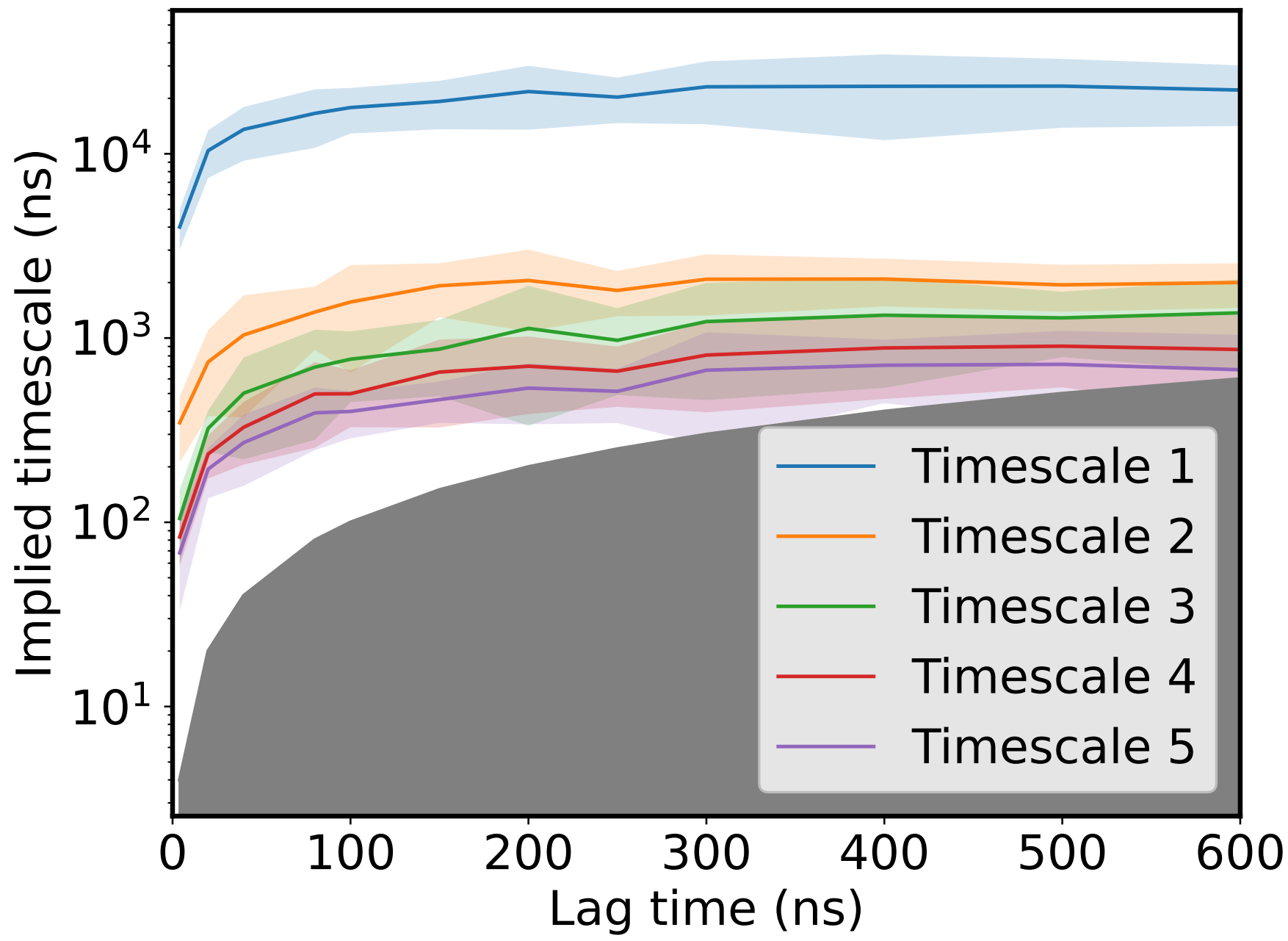

Figure S13 Implied timescale (ITS) convergence plot for an MSM built with regular space clustering on our NTL9 Koopman projected data. We find that a lagtime of 150 ns is sufficient to enable evaluation of converged timescales. Standard errors were measured by bootstrapping; specifically block sub trajectories where sampled randomly with blocks of size 20µs to ensure decorrelation. Error bars which are ± the standard error are plotted

References

(1) Trendelkamp-Schroer, B.; Wu, H.; Paul, F.; Noé, F. Estimation and uncertainty of reversible Markov models. *J. Chem. Phys.* **2015**, *143*.

(2) Prinz, J.-H.; Wu, H.; Sarich, M.; Keller, B.; Senne, M.; Held, M.; Chodera, J. D.; Schütte, C.; Noé, F. Markov models of molecular kinetics: Generation and validation. *J. Chem. Phys.* **2011**, *134*.

(3) Noé, F.; Doose, S.; Daidone, I.; Löllmann, M.; Sauer, M.; Chodera, J. D.; Smith, J. C. Dynamical fingerprints for probing individual relaxation processes in biomolecular dynamics with simulations and kinetic experiments. *Proc. Natl. Acad. Sci. U.S.A.* **2011**, *108*, 4822–4827.

(4) Chodera, J. D.; Swope, W. C.; Pitera, J. W.; Dill, K. A. Long-time protein folding dynamics from short-time molecular dynamics simulations. *Multiscale Model. Simul.* **2006**, *5*, 1214–1226.

(5) Nuske, F.; Keller, B. G.; Pérez-Hernández, G.; Mey, A. S.; Noé, F. Variational approach to molecular kinetics. *J. Chem. Theory Comput.* **2014**, *10*, 1739–1752.

(6) Dellnitz, M.; Junge, O. On the approximation of complicated dynamical behavior. *SIAM J. Numer. Anal.* **1999**, *36*, 491–515.

(7) Bittracher, A.; Koltai, P.; Junge, O. Pseudogenerators of spatial transfer operators. *SIAM J. Appl. Dyn. Syst.* **2015**, *14*, 1478–1517.

(8) Noé, F.; Nuske, F. A variational approach to modeling slow processes in stochastic dynamical systems. *Multiscale Model. Simul.* **2013**, *11*, 635–655.

(9) Wu, H.; Noé, F. Variational approach for learning Markov processes from time series data. *J. Nonlinear Sci.* **2020**, *30*, 23–66.

(10) Huang, J.; MacKerell Jr, A. D. CHARMM36 all-atom additive protein force field: Validation based on comparison to NMR data. *J. Comput. Chem.* **2013**, *34*, 2135–2145.

(11) Jo, S.; Kim, T.; Iyer, V. G.; Im, W. CHARMM-GUI: a web-based graphical user interface for CHARMM. *J. Comput. Chem.* **2008**, *29*, 1859–1865.

(12) Bussi, G.; Donadio, D.; Parrinello, M. Canonical sampling through velocity rescaling. *J. Chem. Phys.* **2007**, *126*.

(13) Bernetti, M.; Bussi, G. Pressure control using stochastic cell rescaling. *J. Chem. Phys.* **2020**, *153*.

(14) Best, R. B.; Hummer, G. Coordinate-dependent diffusion in protein folding. *Proc. Natl. Acad. Sci. U.S.A.* **2010**, *107*, 1088–1093.

(15) Plotkin, S. S.; Wolynes, P. G. Non-Markovian configurational diffusion and reaction coordinates for protein folding. *Phys. Rev. Lett.* **1998**, *80*, 5015.

(16) Dalton, B. A.; Ayaz, C.; Kiefer, H.; Klimek, A.; Tepper, L.; Netz, R. R. Fast protein folding is governed by memory-dependent friction. *Proc. Natl. Acad. Sci. U.S.A.* **2023**, *120*, e2220068120.

(17) Fakhoury, Z.; Sosso, G. C.; Habershon, S. Contact-Map-Driven Exploration of Heterogeneous Protein-Folding Paths. *J. Chem. Theory Comput.* **2024**, *20*, 8340–8353.
